# Healing, surviving, or dying? – projecting the German future disease burden using a Markov illness-death model

**DOI:** 10.1186/s12889-020-09941-6

**Published:** 2021-01-11

**Authors:** Valeska Milan, Stefan Fetzer, Christian Hagist

**Affiliations:** 1 AOK Baden-Württemberg, Stuttgart / WHU Otto Beisheim School of Management, Burgplatz 2, 56179 Vallendar, Germany; 2 Hochschule Aalen – Technik und Wirtschaft, Aalen, Germany; 3 WHU Otto Beisheim School of Management, Vallendar, Germany

**Keywords:** Demography, Projection, Markov illness-death model, Chronic diseases, Compression of morbidity

## Abstract

**Background:**

In view of the upcoming demographic transition, there is still no clear evidence on how increasing life expectancy will affect future disease burden, especially regarding specific diseases. In our study, we project the future development of Germany’s ten most common non-infectious diseases (arthrosis, coronary heart disease, pulmonary, bronchial and tracheal cancer, chronic obstructive pulmonary disease, cerebrovascular diseases, dementia, depression, diabetes, dorsal pain and heart failure) in a Markov illness-death model with recovery until 2060.

**Methods:**

The disease-specific input data stem from a consistent data set of a major sickness fund covering about four million people, the demographic components from official population statistics. Using six different scenarios concerning an expansion and a compression of morbidity as well as increasing recovery and effective prevention, we can show the possible future range of disease burden and, by disentangling the effects, reveal the significant differences between the various diseases in interaction with the demographic components.

**Results:**

Our results indicate that, although strongly age-related diseases like dementia or heart failure show the highest relative increase rates, diseases of the musculoskeletal system, such as dorsal pain and arthrosis, still will be responsible for the majority of the German population’s future disease burden in 2060, with about 25–27 and 13–15 million patients, respectively. Most importantly, for almost all considered diseases a significant increase in burden of disease can be expected even in case of a compression of morbidity.

**Conclusion:**

A massive case-load is emerging on the German health care system, which can only be alleviated by more effective prevention. Immediate action by policy makers and health care managers is needed, as otherwise the prevalence of widespread diseases will become unsustainable from a capacity point-of-view.

## Background

The development of future patient numbers is an important concern for many stakeholders in the health systems. Rational decisions about the planning of hospital capacities, pharmaceutical investments, career choices of (future) healthcare professionals as well as the development of future health care expenditures itself depend on the precise knowledge of the future development of specific diseases.

Germany is one of the fastest ageing countries in the world due to constantly low fertility rates since the 1970s and a continuously increasing life expectancy. In the literature there are different rival theories and hypotheses how an increasing life expectancy will particularly affect the disease burden and the related health care expenditure. Gruenberg (1977) [1] and Verbrugge (1984) [2] hypothesise that a rising longevity goes hand in hand with an increase in years spent in illness and therefore with an *expansion of morbidity* in older age groups. In contrast, Fries (1980) [3] assumes that an increasing life expectancy leads to a *compression of morbidity*. Given these somehow contradictory hypotheses, the influence of proximity to death and treatment spending as a function of remaining life expectancy are controversially discussed among health economists [4–7].

However, even less evidence exists today concerning the (more epidemiological) question of specific diseases’ future development in the light of the different hypotheses. A systematic literature review on PubMed searching for projections (or synonyms) in context of demography and using the keywords *prevalence*, *incidence* or *burden of disease* for specific or chronic non-infectious diseases in general shows 160 relevant publications. There are three categories of studies by their projection methodology: trend extrapolations (99/160), multistate models (57/160) and studies using both methodologies (4/160). In 54 of the studies using trend extrapolation (103/160) indeed current prevalence or incidence rates are transferred to population projections, which excludes a specific modelling of the various theses. This so-called status quo analysis is also commonly used in projections of health expenditures^1^. Out of the 61 studies using multistate modelling (61/160), 17 (17/61) are based on the classical structure of an illness-death model (even if only 7 explicitly define it that way). However, only nine of the studies (9/61) focus on an explicit modelling of a compression of morbidity, of them eight (8/9) related to dementia. Furthermore, just seven studies (7/61) compare the development of more than two different diseases, only one of them modelling compression scenarios [9] (see the appendix for more detailed information and results on the systematic database search).

In our paper, we present projections for ten common non-infectious diseases (arthrosis, coronary heart disease, pulmonary, bronchial and tracheal cancer, chronic obstructive pulmonary disease, cerebrovascular diseases, dementia, depression, diabetes, dorsal pain and heart failure). The selected diseases represent the intersection between the most common and most expensive disease patterns in Germany [10]. For the projections we use a time-discrete Markov illness-death model with recovery. Our model allows us to regard the different hypotheses in context of demographic transition and to quantify the influence of potentially changing variables (disease-specific survival, incidence and recovery rate) on the future frequency of diseases. In addition, we show the influence of successful prevention on long-term prevalence of the different diseases.

The population-related components used for modelling stem from Destatis, the German Federal Statistical Office, whereas the disease-specific components are computed on the data of a major sickness fund covering approximately four million insureds during the period from 2009 to 2017. Our data set is unique as we calculated the input data ourselves using disease-specific validation criteria selected for this purpose (shown in section Dataset). Hence, our study is one of the few that use insurance data (7/160), although the resulting treatment prevalence is of particular importance for decision makers and payers in the health care system. Data sources from other studies of the systematic literature review are surveys or other epidemiological studies (61/160), a literature review for the different input factors (34/160), registries (28/160) or mixed data sources (30/160).

The paper is organised as follows: we start with the presentation of our time-discrete Markov illness-death model with recovery as well as our data set. Then, we show our results for the future development of the ten diseases (average prevalence rates and number of patients) in different populations and scenarios, also considering the results of other publications. This is followed by a discussion of the results in view of the current state of research and the limitations, finishing with a concluding summary.

## Methods

### Markov illness-death model with recovery

We will calculate the future number of patients and the future average prevalence rates for the total population from 2018 to 2060^2^ using a time-discrete Markov illness-death model with recovery. The model is based on the cohort-component-method [11], which is widely used for (official) population projections. Regarding epidemiologic modelling, it can be attributed to the work of Fix & Neyman (1951) [12] and is closely related to those of Manton et al. (1984), Brookmeyer et al. (1998), Brinks et al. (2012), and Andersson et al. (2015) [13–16], but differs inthe detail level of the rich routine data set used. The specific cohort data by age and gender with corresponding detail diagnosis allows us to vary different variables over time (future development of the disease-specific survival rate, incidence rate and recovery rate). In contrast to most other studies using an illness-death approach (16/17) including the work of Milan & Fetzer (2019) [17], on which our modelling is based, the model also includes the possibility of recovery.

The starting point of our model is the number of patients ***P***_***a,g***_ (differentiated by age ***a*** between 0 and 100 and gender ***g*** which is men or women) in our starting year ***T***. It results from the prevalence rate ***p***_***a,g,T***_ multiplied by the cohort size ***K***_***a,g,T***_.

1 $$ {\boldsymbol{P}}_{\boldsymbol{a},\boldsymbol{g},\boldsymbol{T}}={\boldsymbol{K}}_{\boldsymbol{a},\boldsymbol{g},\boldsymbol{T}}{\boldsymbol{p}}_{\boldsymbol{a},\boldsymbol{g},\boldsymbol{T}} $$

In models extrapolating current prevalence rates (status quo analysis) ***p***_***a,g,T***_ is assumed to be constant over time and only the future cohort sizes determine the future development of patients. In contrast to this, for all following years, age- and gender-specific incidence and recovery rates as well as the mortality rates of patients are used in our model to calculate the (future) number of patients ***P***_***a,g,T + t***_**. **At this point we distinguish between the group of patients which are comprised of the surviving patients of the previous year ${\boldsymbol{D}}_{\boldsymbol{a},\boldsymbol{g},\boldsymbol{T}+\boldsymbol{t}}^{\boldsymbol{T}+\boldsymbol{t}-\mathbf{1}}$ and the group of newly diseased patients ***I***_***a,g,T + t***_.

2 $$ {\boldsymbol{P}}_{\boldsymbol{a},\boldsymbol{g},\boldsymbol{T}+\boldsymbol{t}}={\boldsymbol{D}}_{\boldsymbol{a},\boldsymbol{g},\boldsymbol{T}+\boldsymbol{t}}^{\boldsymbol{T}+\boldsymbol{t}-\mathbf{1}}+{\boldsymbol{I}}_{\boldsymbol{a},\boldsymbol{g},\boldsymbol{T}+\boldsymbol{t}} $$

In order to calculate the surviving patients of the previous year ${\boldsymbol{D}}_{\boldsymbol{a},\boldsymbol{g},\boldsymbol{T}+\boldsymbol{t}}^{\boldsymbol{T}+\boldsymbol{t}-\mathbf{1}}$ we use the disease-specific mortality difference ***md***_***a −*** **1*,g,T + t −*** **1**_ which is subtracted from the survival rate of each cohort ***sr***_***a −*** **1*,g,T + t −*** **1**_^3^. Also we consider disease-specific recovery rates ***r***_***a −*** **1*,g,T + t −*** **1**_ as follows^4^:

3 $$ {\boldsymbol{D}}_{\boldsymbol{a},\boldsymbol{g},\boldsymbol{T}+\boldsymbol{t}}^{\boldsymbol{T}+\boldsymbol{t}-\mathbf{1}}={\boldsymbol{P}}_{\boldsymbol{a}-\mathbf{1},\boldsymbol{g},\boldsymbol{T}+\boldsymbol{t}-\mathbf{1}}\left({\boldsymbol{sr}}_{\boldsymbol{a}-\mathbf{1},\boldsymbol{g},\boldsymbol{T}+\boldsymbol{t}-\mathbf{1}}-{\boldsymbol{md}}_{\boldsymbol{a}-\mathbf{1},\boldsymbol{g},\boldsymbol{T}+\boldsymbol{t}-\mathbf{1}}\right)\left(\mathbf{1}-{\boldsymbol{r}}_{\boldsymbol{a}-\mathbf{1},\boldsymbol{g},\boldsymbol{T}+\boldsymbol{t}-\mathbf{1}}\right) $$

To determine the number of new patients ***I***_***a***, ***g***, ***T*** + ***t***_, the number of surviving non-diseased from the previous year is calculated as follows in a first step:

4 $$ {\boldsymbol{ND}}_{\boldsymbol{a},\boldsymbol{g},\boldsymbol{T}+\boldsymbol{t}}^{\boldsymbol{T}+\boldsymbol{t}-\mathbf{1}}={\boldsymbol{K}}_{\boldsymbol{a}-\mathbf{1},\boldsymbol{g},\boldsymbol{T}+\boldsymbol{t}-\mathbf{1}}{\boldsymbol{sr}}_{\boldsymbol{a}-\mathbf{1},\boldsymbol{g},\boldsymbol{T}+\boldsymbol{t}-\mathbf{1}}-{\boldsymbol{D}}_{\boldsymbol{a},\boldsymbol{g},\boldsymbol{T}+\boldsymbol{t}}^{\boldsymbol{T}+\boldsymbol{t}-\mathbf{1}} $$

In a second step the number of new patients ***I***_***a,g,T + t***_, which results from the age- and gender-specific incidence rate ***i***_***a,g,T + t***_, is multiplied with the surviving non-diseased from the previous year:

5 $$ {\boldsymbol{I}}_{\boldsymbol{a},\boldsymbol{g},\boldsymbol{T}+\boldsymbol{t}}={\boldsymbol{ND}}_{\boldsymbol{a},\boldsymbol{g},\boldsymbol{T}+\boldsymbol{t}}^{\boldsymbol{T}+\boldsymbol{t}-\mathbf{1}}{\boldsymbol{i}}_{\boldsymbol{a},\boldsymbol{g},\boldsymbol{T}+\boldsymbol{t}} $$

The total number of patients ***P***_***T + t***_ in all years ***T + t*** is finally calculated as:

6 $$ {\boldsymbol{P}}_{\boldsymbol{T}+\boldsymbol{t}}={\sum}_{\boldsymbol{a}=\mathbf{0}}^{\mathbf{100}}\left({\boldsymbol{D}}_{\boldsymbol{a},\boldsymbol{women},\boldsymbol{T}+\boldsymbol{t}}^{\boldsymbol{T}+\boldsymbol{t}-\mathbf{1}}+{\boldsymbol{I}}_{\boldsymbol{a},\boldsymbol{women},\boldsymbol{T}+\boldsymbol{t}}\right)+{\sum}_{\boldsymbol{a}=\mathbf{0}}^{\mathbf{100}}\left({\boldsymbol{D}}_{\boldsymbol{a},\boldsymbol{men},\boldsymbol{T}+\boldsymbol{t}}^{\boldsymbol{T}+\boldsymbol{t}-\mathbf{1}}+{\boldsymbol{I}}_{\boldsymbol{a},\boldsymbol{men},\boldsymbol{T}+\boldsymbol{t}}\right) $$

In our model for all years ***T + t*** the future cohort sizes, ***K***_***a,g,T + t***_ as well as the future survival rates ***sr***_***a,g,T + t***_ of the total population are derived from a population projection, which we calculate via the cohort component method. Within this framework we consider the disease-specific components. The calculation of the survival rate of the patients as the difference ***sr***_***a −*** **1*,g,T + t −*** **1**_ − ***md***_***a −*** **1*,g,T + t −*** **1**_ and the surviving non-diseased ${\boldsymbol{ND}}_{\boldsymbol{a},\boldsymbol{g},\boldsymbol{T}+\boldsymbol{t}}^{\boldsymbol{T}+\boldsymbol{t}-\mathbf{1}}$ as the difference between all survivors of the cohort and the surviving patients from the previous period finally merge the population projection with the epidemiological developments. Thus, the design of our model also allows the use of input data from any other population projection or/and disease-specific statistic. This time-discrete approach is also more intuitive to understand for a broader audience, such as policy setters and health care decision makers.

Dividing the total number of patients by the total number of the population results in the average prevalence rate of the total population, ***apr***, which we will present in addition to the total number of patients in the result section. Obviously, the ***apr*** highly depends on the share of the elderly and diseased within the total population. As the German demographic transition leads to an increasing proportion of elderly cohorts, we call this effect *cohort effect*, which can also be observed in models extrapolating current prevalence rates using the status quo analysis.

As for the further effects of our model, we will take a closer look at the future age- and gender-related prevalence rate ***p***_***T +*** **1**_, which can be obtained by dividing the number of patients (eqs. 2 to 5) by the total corresponding cohort ***K***_***a,g,T + t***_ = ***K***_***a −*** **1*,g,T + t −*** **1**_***sr***_***a −*** **1*,g,T + t −*** **1**_ and therefore is independent of future cohort sizes:

7 $$ {\boldsymbol{p}}_{\boldsymbol{T}+\mathbf{1}}=\frac{{\boldsymbol{p}}_{\boldsymbol{T}}\left(\mathbf{1}-\boldsymbol{i}\right)\left(\mathbf{1}-\boldsymbol{r}\right)\left(\boldsymbol{sr}-\boldsymbol{md}\right)+\boldsymbol{isr}}{\boldsymbol{sr}} $$

For reasons of simplicity we use time-independent incidence, recovery and mortality rates and abstract from the indices of age and gender in eq. (7). The total derivate can be used to determine the impact of changing incidence, recovery and mortality rates on the prevalence in year ***T*** + **1**.

$$ {\boldsymbol{dp}}_{\boldsymbol{T}+\mathbf{1}}=\left(\left(\mathbf{1}-\boldsymbol{i}\right)\left(\mathbf{1}-\boldsymbol{r}\right)\frac{\left(\boldsymbol{sr}-\boldsymbol{md}\right)}{\boldsymbol{sr}}\right)\boldsymbol{d}{\boldsymbol{p}}_{\boldsymbol{T}} $$

$$ +\left(\frac{{\boldsymbol{P}}_{\boldsymbol{T}}\left(\mathbf{1}-\boldsymbol{i}\right)\left(\mathbf{1}-\boldsymbol{r}\right)+\boldsymbol{i}-{\boldsymbol{p}}_{\boldsymbol{T}+\mathbf{1}}}{\boldsymbol{sr}}\right)\boldsymbol{dsr} $$

$$ -\left(\frac{{\boldsymbol{P}}_{\boldsymbol{T}}\left(\mathbf{1}-\boldsymbol{i}\right)\left(\mathbf{1}-\boldsymbol{r}\right)}{\boldsymbol{sr}}\right)\boldsymbol{dmd} $$

$$ -\left({\boldsymbol{P}}_{\boldsymbol{T}}\left(\mathbf{1}-\boldsymbol{i}\right)\frac{\left(\boldsymbol{sr}-\boldsymbol{md}\right)}{\boldsymbol{sr}}\right)\boldsymbol{dr} $$

8 $$ +\left(\mathbf{1}-{\boldsymbol{P}}_{\boldsymbol{T}}\frac{\left(\boldsymbol{sr}-\boldsymbol{md}\right)}{\boldsymbol{sr}}+{\boldsymbol{P}}_{\boldsymbol{T}}\boldsymbol{hr}\frac{\left(\boldsymbol{sr}-\boldsymbol{md}\right)}{\boldsymbol{sr}}\right)\boldsymbol{di} $$

In our model specification, the variables ***p***_***T***_***, sr, md, r*** and ***i*** can take on values between 0 and 1 and the disease-specific mortality difference ***md*** is less (or in theory equal) than the survival rate of the entire population ***sr***. As eq. (8) shows, a higher prevalence rate ***p*** in year ***T*** leads to a higher prevalence rate in year ***T +*** **1**. The theoretical one-to-one impact of this effect is lowered by the degree of the incidence and recovery rate as well as the disease-specific mortality difference.

An increase of the survival rate ***sr*** initially leads to an increase in both, the diseased and the non-diseased population. In conjunction with the incidence rate ***i***, a positive impact on the prevalence rate in year ***T +*** **1** can be observed as the rising survival rate leads to a higher “at risk” population. In contrast to this, a higher mortality difference ***md*** leads to a decline in the prevalence rate in year ***T +*** **1**. Both effects combined can be interpreted as follows: The smaller the difference in mortality between the diseased and non-diseased, the higher the positive impact of an increasing survival rate.

The influence of the recovery rate is negative and linked to the life expectancy of the patients. The more patients survive until the following year, the more can recover again. However, the higher the incidence rate and thus the proportion of new patients, the lower the proportion of persons who could potentially recover, which mitigates the negative effect of the recovery rate.

Considering the impact of increasing incidence rates also offers a connection between the incidence and the recovery rate. A higher proportion of recovered people leads to a higher “at-risk” population. The opposite effect results from a higher prevalence rate in year ***T*** which comes along with a lower “at-risk” population.

### Scenarios

Regarding the effects outlined above, a change of one variable will always affect the future prevalence in interaction with the other components. To illustrate these effects and the sensitivity of the model, we model six scenarios of changing disease-specific variables ***md***_***a,g***_***, i***_***a,g***_ and ***r***_***a,g***_ for each of the ten diseases up to 2060, especially regarding the different hypotheses of expansion and compression of morbidity (see Table 1). In all scenarios we assume increasing survival rates ***sr***_***a,g***_ according to the moderately increasing life expectancy scenario *L2* [18]).

**Table 1:** Scenarios, assumptions and their effect on the future prevalence rate

		Variables				Effect on ***dp***_***T +*** **1**_	Implementation
		***md***	***sr***	***i***	***r***		
**Expansion 1** (Exp1)	Scenario		**+**			**+**	Increasing ***sr***_***a,g***_ according to *L2* scenario
	Effect		**+**				
**Expansion 2** (Exp2)	Scenario		**+**	**+**		**++**	Increasing ***sr***_***a,g***_ according to L2 scenario Linearly increasing ***i*** of 30% until 2060
	Effect	**+**	**+**				
**Compression 1** (Comp1)	Scenario	**+**	**+**			**?**	Increasing ***sr***_***a,g***_ according to *L2* scenario Increasing ***md***_***a,g***_ corresponding to increasing ***sr***
	Effect	**–**	**+**				
**Compression 2** (Comp2)	Scenario		**+**	**–**		**?**	Increasing ***sr***_***a,g***_ according to *L2* scenario Shift of ***i***_***a,g***_ corresponding to increasing ***sr***_***a,g***_ resulting in a continuous decrease of ***i***
	Effect		**+**	**–**			
**Prevention** (Prev)	Scenario		**+**	**–** **–**		**?**	Increasing ***sr***_***a,g***_ according to *L2* scenario Linearly decreasing ***i***_***a,g***_ of 30% until 2035
	Effect		**+**	**–** **–**			
**Extended Recovery** (Rec)	Scenario		**+**		**+**	**?**	Increasing ***sr***_***a,g***_ according to *L2* scenario Linearly increasing ***r***_***a,g***_ of 50% until 2060
	Effect		**+**		**–**		

Source: Own depiction

In the first scenario, we hold all disease-specific variables constant over the time horizon. However, the assumption of a constant mortality difference and rising survival rates (***sr***_***a,g,T + t***_ ***> sr***_***a,g,T + t −*** **1**_) leads to an increase in life expectancy of both the non-diseased and the diseased. In conjunction with constant incidence rates (***i***_***ag***_ ***= const***), this results in an increasing duration of disease. Thus, the scenario *Expansion 1* can be interpreted as a type of *expansion of morbidity hypothesis*. This scenario serves as our baseline scenario in the following. The scenario *Expansion* 2 is a more extreme scenario of the *expansion of morbidity hypothesis*, assuming an additional 30% increase in incidence rates until 2060 (***i***_***a,g,T + t***_ ***> i***_***a,g,T + t −*** **1**_).

The *compression of morbidity hypothesis *is considered in two different scenarios: In the scenario *Compression 1 *only the healthy population benefits from the increasing life expectancy (${\boldsymbol{sr}}_{\boldsymbol{a},\boldsymbol{g}}^{\boldsymbol{D}}=\boldsymbol{const}$) which leads to a continuous increase in the mortality difference between the diseased and the healthy population. In the scenario *Compression 2* a shift of diseased cases in relation to increasing life expectancy is modelled which is in line with the “traditional” *compression of morbidity hypothesis* and leads to continuously decreasing incidence rates (***i***_***a,g,T + t***_ ***< i***_***a,g,T + t −*** **1**_).

To highlight the long-term impact of effective prevention programmes, a scenario *Prevention *is modelled with temporarily decreasing incidence rates (***i***_***a,g,T + t***_ ***< i***_***a,g,T + t −*** **1**_) up to 30% until 2035. In order to simulate possible effects of better medical care, e.g. due to disease management programmes, the scenario *Extended Recovery* assumes increasing recovery rates up to 50% until the year 2060 (***r***_***a,g,T + t***_ ***> r***_***a,g,T + t −*** **1**_).

Interestingly (and as discussed in the section on the total differential of the prevalence rate), the total effect of the scenarios *Compression 1* and *2* as well as of the scenarios *Extended Recovery* and *Prevention* on the future (age- and gender-related) prevalence rate is not defined a priori and depends on the numerical ratio of disease-related input data and the increase of survival rates.

### Dataset

The average disease-specific input data for each cohort and gender^5^ derives from a routine dataset of around four million insureds of the AOK Baden-Württemberg from 2009 to 2017^6^. Due to this large number of people insured by the AOK in Baden-Württemberg, this population is approximately representative of the German population regarding the disease-rates within the age cohorts. Table 2 shows the specific selection criteria for each of the ten diseases. Since there are no coding guidelines for outpatient diagnoses in Germany, we use the criteria of the AOK Research Institute published in various reports [19–22]. The M2Q^7^/M3Q criterion, for instance, only defines patients as diseased if they have a confirmed diagnosis in at least two and three out of four quarters of the year, respectively. Inpatient primary and secondary diagnosis are included without additional validation criteria. We complete missing data by the following procedure: If the selection criteria are satisfied the year before and the year after, insureds are classified as patients also in the incompletely coded year. Patients are classified as “new patients” when they fail to fulfil the prevalence criteria in any of the four previous years. The days of insurance of the patients identified by diagnosis are then set in relation to those of all insureds to calculate period prevalence ***p***_***a,g***_ and cumulative incidence ***i***_***a,g***_ for the years 2015 to 2017 [24]. For pulmonary cancer we use a five-year pre-observation period for the derivation of the incidence. To take into account the periodic character of depression, we use additional selection criteria for new cases and divergent diagnoses to determine prevalence and incidence.^8^

For the calculation of recovery rates ***r***_***a,g***_ all surviving patients without a coded diagnosis in the following years are set in relation to the total of all surviving patients. For the definition of recovery we use a four-year follow-up period for diseases with realistic cure probabilities (dorsal pain, depression and CVD) and a five-year follow-up period for pulmonary cancer. The maximum follow-up period of 8 years is used for all other diseases since there are still no cure possibilities available for their most common manifestations. Since dementia is (as of yet) characterized by an irreversible disease progression, no recovery rates are considered in these calculations^9^. For chronic diseases, the recovery rates are to be interpreted as being symptom-free. A recurrence of the disease after years of asymptomatic illness is taken into account by the incidence rate. For each cohort, we calculate mortality differences ***md***_***a,g***_ as the difference between the 1-year survival rates of the diseased and all insureds in a given year and subtract them from the German population’s survival probability ***sr***_***a,g***_ as described above^10^. Table 3 shows the population weighted determined input data as the average value for different age groups and overall average in the base year 2018 for each disease, in parentheses differentiated by gender (female vs male). In addition, Table 4 illustrates the demographic characteristics of the study population as average values of all years analyzed in millions and as percentage compared to those of the entire German population in 2018.^11^

**Table 3:** Determined disease-specific variables

		Age cohort				
	Total	18–29	30–44	45–64	65–84	> 85
**German population**						
Million	83.0 (5.5, 6.0)	11.5 (7.6. 7.9)	15.5 (12.3, 12.3)	24.6 (8.5, 7.1)	15.6 (8.5, 7.1)	2.3 (1.5, 0.7)
%		13.8 (6.6, 7.2)	18.7 (9.2, 9.5)	29.7 (14.8, 14.8)	18.9 (10.3, 8.5)	2.8 (1.8, 0.9)
**Study population**						
Million	4.0 (2.1, 1.9)	0.6 (0.3, 0.3)	0.7 (0.4, 0.3)	1.1 (0.6, 0.5)	0.8 (0.4, 0.3)	0.13 (0.09, 0.03)
%		15.3 (7.6, 7.7)	17.9 (9.1, 8.8)	27.8 (14.4, 13.4)	19.4 (11.1, 8.3)	3.1 (2.3, 0.8)
**Life expectancy total population**	98.89 (98.90, 98.88)	99.97 (99.98, 99.96)	99.92 (99.94, 99.90)	99.51 (99.65, 99.37)	97.11 (97.67, 96.43)	85.51 (86.08, 84,31)
**Arthrosis**						
***p*** (%)	13.4 (15.8, 10.8)	0.4 (0.3, 0.4)	2.1 (2.1, 2.1)	15.6 (17.6, 13.5)	37.3 (41.6, 32.1)	46.9 (49.5, 41.5)
***i*** (%)	1.5 (1.6, 1.3)	0.2 (0.2, 0.2)	0.7 (0.7, 0.7)	2.6 (2.8, 2.3)	2.7 (2.8, 2.5)	2.1 (2.0, 2.2)
***r*** (% of patients)	1.6 (1.4, 1.8)	11.5 (12.4, 10.8)	6.3 (6.0, 6.6)	2.2 (2.0, 2.5)	1.0 (1.0, 1.1)	0.2 (0.2, 0.2)
***md*** (%-points)	–	–	–	–	–	–
**CA**						
***p*** (%)	0.20 (0.14, 0.26)	0.00 (0.00, 0.00)	0.01 (0.01, 0.01)	0.22 (0.17, 0.27)	0.63 (0.39, 0.92)	0.35 (0.21, 0.63)
***i*** (%)	0.07 (0.05, 0.09)	0.00 (0.00, 0.00)	0.01 (0.01, 0.01)	0.08 (0.06, 0.11)	0.21 (0.13, 0.32)	0.12 (0.07, 0.22)
***r*** (% of patients)	1.9 (2.5, 1.6)	–	0.5 (0.7, 0.2)	2.2 (2.7, 1.9)	1.8 (2.4, 1.4)	1.8 (2.2, 1.5)
***md*** (%-points)	24.4 (22.0, 25.7)	–	14.9 (14.2, 15.8)	23.4 (20.9, 25.0)	24.8 (22.7, 25.8)	29.1 (25.1, 31.9)
**CHD**						
***p*** (%)	5.9 (4.5, 7.3)	0.0 (0.0, 0.0)	0.3 (0.1, 0.5)	4.8 (2.5, 7.2)	19.2 (14.1, 25.4)	29.2 (25.8, 36.4)
***i*** (%)	0.7 (0.6, 0.8)	0.0 (0.0, 0.0)	0.1 (0.1, 0.2)	0.8 (0.5, 1.0)	1.9 (1.6, 2.1)	2.0 (1.9, 2.1)
***r*** (% of patients)	1.4 (2.0, 1.0)	–	8.3 (13.2, 6.9)	2.7 (5.2, 1.8)	1.0 (1.7, 0.6)	0.2 (0.3, 0.1)
***md*** (%-points)	1.3 (1.3, 1.2)	–	1.1 (1.6, 0.9)	1.0 (1.0, 1.0)	1.4 (1.5, 1.4)	0.9 (0.9, 0.9)
**COPD**						
***p*** (%)	4.1 (3.6, 4.6)	0.3 (0.3, 0.3)	0.9 (0.9, 1.0)	4.9 (4.2, 5.6)	10.8 (8.7, 13.3)	11.3 (9.1, 15.7)
***i*** (%)	0.5 (0.4, 0.5)	0.1 (0.1, 0.1)	0.2 (0.2, 0.2)	0.7 (0.6, 0.8)	1.0 (0.8, 1.2)	0.9 (0.8,1.1)
***r*** (% of patients)	2.5 (2.9, 2.2)	7.8 (8.3, 7.3)	6.3 (6.5, 6.1)	2.8 (3.2, 2.5)	1.6 (2.2, 1.2)	0.4 (0.6, 0.2)
***md*** (%-points)	1.4 (1.2, 1.7)	0.0 (0.0, 0.0)	0.3 (0.1, 0.4)	0.9 (0.7, 1.1)	2.0 (1.6, 2.3)	1.7 (1.2, 2.2)
**CVD**						
***p*** (%)	4.6 (4.4, 4.8)	0.1 (0.1, 0.1)	0.4 (0.4, 0.4)	3.2 (2.7, 3.8)	15.1 (13.3, 17.4)	25.5 (24.2, 28.3)
***i*** (%)	0.7 (0.7, 0.7)	0.0 (0.0, 0.0)	0.1 (0.1, 0.1)	0.7 (0.6, 0.8)	2.2 (2.0, 2.4)	3.0 (3.0, 3.0)
***r*** (% of patients)	2.9 (3.2, 2.5)	8.9 (9.0, 8.9)	6.7 (7.3, 6.0)	4.1 (3.1, 3.7)	2.6 (3.1, 2.2)	1.7 (1.9, 1.3)
***md*** (%-points)	1.4 (1.4, 1.4)	1.2 (1.1, 1.3)	1.4 (1.2, 1.6)	1.1 (1.0, 1.3)	1.5 (1.5, 1.5)	1.6 (1.7, 1.3)
**Dementia**						
***p*** (%)	2.0 (2.4, 1.6)	0.0 (0.0, 0.0)	0.0 (0.0, 0.0)	0.4 (0.3, 0.5)	6.3 (6.3, 6.2)	26.4 (28.1, 22.9)
***i*** (%)	0.4 (0.5, 0.4)	0.0 (0.0, 0.0)	0.0 (0.0, 0.0)	0.1 (0.1, 0.1)	1.4 (1.4, 1.5)	4.8 (4.9, 4.7)
***r*** (% of patients)	–	–	–	–	–	–
***md*** (%-points)	6.0 (5.2, 7.2)	–	1.5 (1.5, 1.5)	3.3 (2.5, 3.9)	6.3 (5.4, 7.3)	5.9 (5.1, 8.1)
**Depression**						
***p*** (%)	12.5 (16.1, 8.7)	5.0 (6.6, 3.6)	9.9 (12.7, 7.2)	17.7 (22.3, 13.1)	20.8 (25.9, 14.7)	24.8 (28.5, 17.1)
***i*** (%)	0.9 (1.0, 0.8)	0.8 (1.1, 0.7)	0.9 (1.1, 0.7)	1.0 (1.1, 1.0)	1.1 (1.2, 1.0)	1.4 (1.5, 1.4)
***r*** (% of patients)	3.2 (2.9, 3.6)	5.8 (5.8, 5.8)	4.1 (4.0, 4.4)	3.1 (2.8, 3.6)	2.6 (2.4, 3.0)	1.3 (1.4, 1.1)
***md*** (%-points)	0.1 (0.0, 0.1)	0.0 (0.0, 0.1)	0.1 (0.0, 0.1)	0.1 (0.0, 0.1)	0.1 (0.0, 0.2)	–
**Diabetes**						
***p*** (%)	11.1 (10.9, 11.2)	0.8 (0.9, 0.6)	2.5 (2.7, 2.3)	12.1 (10.4, 13.8)	31.6 (29.6, 34.1)	34.3 (34.2, 34.5)
***i*** (%)	0.7 (0.7, 0.7)	0.2 (0.3, 0.1)	0.5 (0.6, 0.4)	1.0 (0.9, 1.2)	1.4 (1.3, 1.5)	1.1 (1.0, 1.2)
***r*** (% of patients)	0.6 (0.7, 0.5)	3.4 (4.8, 1.4)	2.1 (3.1, 1.0)	0.7 (0.7, 0.6)	0.4 (0.5, 0.4)	0.2 (0.2, 0.1)
***md*** (%-points)	0.4 (0.4, 0.4)	0.1 (0.1, 0.2)	0.2 (0.1, 0.3)	0.2 (0.1, 0.3)	0.4 (0.4, 0.4)	0.3 (0.3, 0.3)
**Dorsal pain**						
***p*** (%)	28.3 (31.7, 24.8)	12.0 (13.8, 10.3)	22.7 (25.3, 20.1)	39.4 (43.3, 35.5)	48.7 (52.1, 44.5)	45.2 (46.5, 42.4)
***i*** (%)	2.5 (2.5, 2.4)	3.0 (3.3, 2.8)	3.3 (3.5, 3.2)	2.8 (2.8, 2.9)	2.1 (2.1, 2.1)	1.6 (1.6, 1.8)
***r*** (% of patients)	1.7 (1.6, 1.9)	3.1 (3.0, 3.1)	2.0 (1.9, 2.2)	1.5 (1.3, 1.7)	1.5 (1.4, 1.6)	1.5 (1.5, 1.3)
***md*** (%-points)	–	–	–	–	–	–
**HF**						
***p*** (%)	4.5 (4.6, 4.4)	0.0 (0.0, 0.1)	0.2 (0.2, 0.3)	2.5 (1.8, 3.2)	14.4 (13.4, 15.7)	35.3 (35.7, 34.5)
***i*** (%)	0.9 (0.9, 0.9)	0.0 (0.0, 0.0)	0.1 (0.1, 0.1)	0.7 (0.5, 0.9)	2.8 (2.6, 3.1)	4.9 (4.6, 5.4)
***r*** (% of patients)	2.6 (2.3, 2.9)	14.1 (15.3, 13.5)	11.4 (13.3, 10.4)	6.9 (7.1, 6.8)	2.1 (2.2, 2.0)	0.2 (0.2, 0.1)
***md*** (%-points)	4.3 (3.7, 4.9)	4.1 (5.0, 3.7)	3.3 (3.6, 3.1)	3.1 (2.9, 3.3)	4.5 (3.8, 5.2)	4.7 (4.0, 6.2)

Source: Own Data and depiction in combination with data of Destatis and mortality.org, in parentheses differentiated by gender (female/male) Abbreviations: *p* prevalence rate, *i* incidence rate, *r* recovery rate, *md* mortality difference, *CA* pulmonary, bronchial and tracheal cancer, *CHD* coronary heart disease, *COPD* chronic obstructive pulmonary disease, *CVD* cerebrovascular diseases, *HF* heart failure

**Table 4:** Projected average prevalence rates ***apr*** 2060 and percentage change compared to 2018

	***apr***		%change for different populations		
	2018	2060	Stationary population (only epidemiology)	Population (LE constant)	***Expansion 1*** Standard Population (LE increasing)
**Arthrosis**	13.4%	22.7%	*30.8%*	*56.0%*	***69.8%***
**CA**	0.2%	0.3%	*16.2%*	*42.2%*	***53.9%***
**CHD**	5.9%	9.5%	*5.0%*	*38.5%*	***60.4%***
**COPD**	4.1%	7.1%	*31.0%*	*56.2%*	***71.9%***
**CVD**	4.6%	8.7%	*22.7%*	*63.2%*	***89.9%***
**Dementia**	2.0%	4.4%	*8.1%*	*67.7%*	***117.9%***
**Depression**	12.5%	14.4%	*3.3%*	*10.1%*	***15.7%***
**Diabetes**	11.1%	14.4%	*0.3%*	*18.8%*	***29.7%***
**Dorsal pain**	28.3%	41.4%	*39.9%*	*43.4%*	***46.1%***
**HF**	4.5%	8.5%	*15.4%*	*60.3%*	***90.4%***

Source: Own depiction

Abbreviations: *CA* pulmonary, bronchial and tracheal cancer, *CHD* coronary heart disease, *COPD* chronic obstructive pulmonary disease, *CVD* cerebrovascular diseases, *HF* heart failure

Inorder to derive the (future) cohort sizes ***K***_***a,g***_ and survival rates ***sr***_***a,g***_**, **we build different population projections based on input data from Destatis and statistics of mortality.org. As our starting point serves a *Stationary Population* with constant absolute births and constant life expectancy to separate the effects resulting from disease-specific (epidemiological) components from the effects of the composition of future cohort sizes on the ***apr***. In our second population projection *Population (LE constant)* we abstract from a further increase in life expectancy. This projection is based on the German population in 2018 under the assumption of a fertility rate of 1.55 children per woman of fertile age. For our third population projection, *Standard Population (LE increasing)*, we further assume an increase of life expectancy from 83.3 to 88.1 years at birth for women and 78.5 to 84.4 at birth for men according to the moderate increase scenario *L2* of the 14th population projection [18]. Migration movement is not taken into account, as too little is known about whether disease rates of the German population are transferrable to migrants [27, 28]. Hence, the *Standard Population (LE increasing)* represents an absolute decline in population from 83.0 to 66.2 million by 2060, accompanied by an increasing old-age dependency ratio from 35.9 to 69.7%.^12^ However, for reason of comparability to other studies, we build a fourth population projection, *Population (Migration)*, where future migration is integrated according to the scenario *W2* of the 14th population projection [18].^13^ In this case the total population is 79.1 million people in 2060 and the old-age dependency is 58.8%.

## Results

The presentation of our results starts in Table 4 with a comparison of the average prevalence rates ***apr*** (i.e. the total number of patients divided by the total number of the population) in the years 2018 and 2060 under the assumption of constant disease-specific variables over the time horizon. We use the three different population projections *Stationary Population*, *Population (LE constant)* and *Standard Population (LE increasing)* to separate the effects resulting from disease-specific (epidemiological) components and those occurring from the demographic components (initial population structure and increasing life expectancy). The values resulting from *Standard Population (LE increasing)* correspond to the baseline scenario *Expansion 1*.

The results show a high increase in the ***apr*** for strongly age-related diseases like dementia, heart failure or CVD, with the ageing of the German population due to its current structure (*Population (LE constant)*) and rising life expectancy being the key factors driving the large growth rates. The ratio of people with dementia could more than double by 2060 within the *Standard Population (LE increasing)*. In contrast, the increase of the ***apr*** of dorsal pain is mainly driven by the epidemiological effect. Regarding arthrosis and COPD, the increase of ***apr*** can be attributed to both, the epidemiological as well as the demographic effects. The smallest increase of ***apr*** emerges for diabetes and depression. For both, the epidemiological effect is comparatively low. However, an increase in the average prevalence rate is to be expected for all diseases given the baseline scenario *Expansion 1*. Even when abstracting from an increasing life expectancy, the ageing of the German population in conjunction with the epidemiological effects will lead to a substantial increase of all diseases.

**Fig. 1:**
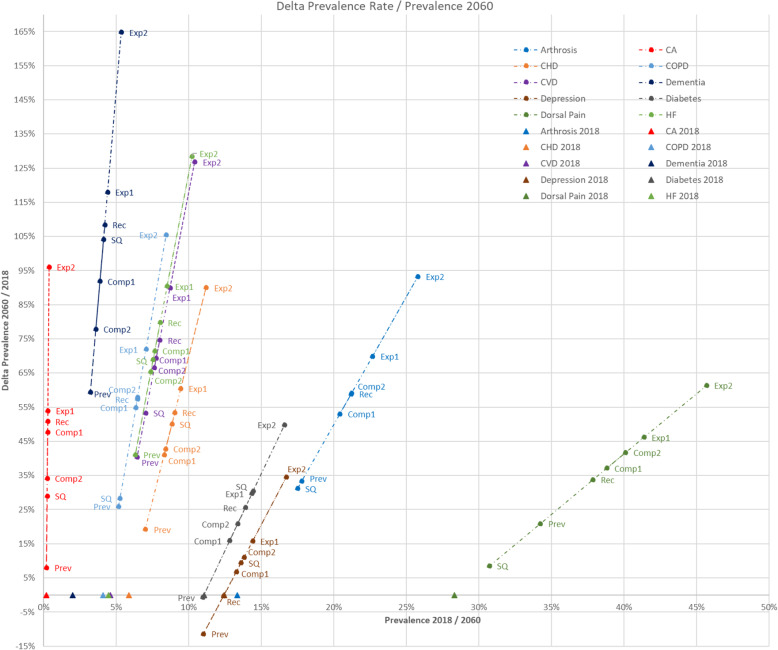
Relative change in *apr* until 2060 in the different scenarios. Source: Own depiction. Abbreviations: Exp1 = scenario Expansion 1, Exp2 = scenario Expansion 2, Comp1 = scenario Compression 1, Comp2 = scenario Compression 2, Rec = scenario Extended Recovery, Prev = scenario Prevention, CA = pulmonary, bronchial and tracheal cancer, CHD = coronary heart disease, COPD = chronic obstructive pulmonary disease, CVD = cerebrovascular diseases, HF = heart failure

Figure 1 presents the results for the ***apr*** in the year 2060 that occur under the different model scenarios (see Table 2) as well as under a simple extrapolation of age- and gender-related prevalence rates for the population of 2060 (status quo (SQ) principle). For this purpose, we use the *Standard Population (LE increasing)*. The y-axis of Fig. 1 shows the relative change of the ***apr*** between 2018 and 2060 whereas the x-axis displays the value of the ***apr*** for the different scenarios in 2060. Additionally, the x-axis depicts the numbers of ***apr*** in 2018.

As a first result, Fig. 1 illustrates that the ranking of the ten diseases with respect to the value of the ***apr*** in 2060 is the same as in 2018, even though the relative change of the ***apr*** differs significantly between the ten diseases. That means that dorsal pain and arthrosis are expected to be the two major diagnoses in 2060, although e.g. dementia offers a significantly higher change in the ***apr*** in all scenarios.

Second, the results show a different impact of the rival hypotheses regarding the consequences of increasing life expectancy on future disease burden: The expansion of morbidity scenarios *Expansion 1* and *2* lead to a soaring increase of all diseases compared to the other scenarios. Especially the scenario of *Expansion 2 *(with an assumed increase of the incidence rate by 30% until 2060) offers a strong increase of the ***apr****. *For strongly age-related diseases such as dementia, CVD or HF, the *Compression 2* scenario (shifting the incidence to higher age groups) has a stronger impact on the ***apr*** than the *Compression 1* scenario, in which the life expectancy for patients is constant over time and only the healthy population benefits from the increasing life expectancy. Yet even in the *compression of morbidity* scenarios, an increase in all the common diseases can be expected. In other words: The increase in burden of disease due to increasing life expectancy and high incidence rates in older age groups can be mitigated but not fully compensated by a compression.

The assumption of continuously rising recovery rates (scenario *Extended Recovery*) has an even smaller impact on future ***apr***, although this is also attributable to the low chances of recovery for the considered diseases in general. Only for depression an increasing recovery rate would lead to a constant prevalence rate in the long term. A diminishing effect on future long-term prevalence for all diseases can only be seen in the scenario *Prevention*. For diabetes and depression, the *Prevention *scenario even leads to a small decline in the ***apr***. This highlights the importance of effective prevention regarding the upcoming demographic transition.

At a first glance a (simple) extrapolation of current prevalence rates should range between the expansion and compression scenarios, our results offer that this is not true for all diseases. In particular, for dorsal pain, arthrosis, COPD, and cancer the *status quo* principle leads to an ***apr*** in 2060 which is smaller than the scenarios of *Prevention*. Hence, our results show a wide range future developments of the different diseases depending on the chosen parameters for modelling.

Table 5 shows the absolute results of the projection for 2040 and 2060. As the *Standard Population (LE increasing)* neglects future migration, the total number of people in Germany will decline between 2040 and 2060. Thus, for the most scenarios and diseases the total numbers of patients are higher in 2040 than 2060. However, the results given the projection *Population (Migration)* in parentheses offer the opposite effect. Hereby we assume identical disease-related input data for migrants.

**Table 5:** Projected number of patients 2060 in the different scenarios

		Number of patients ***P*** (million) in 2060											
		Expansion 1		Expansion 2		Compression 1		Compression 2		Extended Recovery		Prevention	
	**2018**	**2040**	**2060**	**2040**	**2060**	**2040**	**2060**	**2040**	**2060**	**2040**	**2060**	**2040**	**2060**
**Arthrosis**	11.1	15.7	15.0	16.7	17.1	15.1	13.5	15.3	14.1	15.3	14.0	13.5	11.8
		*(16.1)*	*(16.5)*	*(17.0)*	*(18.8)*	*(15.5)*	*(14.9)*	*(15.6)*	*(15.3)*	*(15.7)*	*(15.4)*	*(13.7)*	*(12.9)*
**CA**	0.16	0.22	0.20	0.25	0.26	0.22	0.19	0.21	0.17	0.22	0.20	0.16	0.14
		*(0.22)*	*(0.22)*	*(0.25)*	*(0.28)*	*(0.22)*	*(0.21)*	*(0.21)*	*(0.19)*	*(0.22)*	*(0.21)*	*(0.16)*	*(0.15)*
**CHD**	4.9	6.4	6.3	6.9	7.4	6.0	5.5	6.1	5.6	6.2	6.0	5.2	4.7
		*(6.4)*	*(6.7)*	*(6.9)*	*(7.9)*	*(6.1)*	*(5.9)*	*(6.1)*	*(5.9)*	*(6.3)*	*(6.4)*	*(5.2)*	*(5.0)*
**COPD**	3.4	4.9	4.7	5.3	5.6	4.7	4.2	4.7	4.3	4.7	4.3	4.0	3.4
		*(5.0)*	*(5.1)*	*(5.4)*	*(6.1)*	*(4.8)*	*(4.6)*	*(4.8)*	*(4.7)*	*(4.8)*	*(4.7)*	*(4.1)*	*(3.7)*
**CVD**	3.8	5.8	5.8	6.3	6.9	5.5	5.2	5.5	5.1	5.6	5.3	4.6	4.3
		*(5.9)*	*(6.2)*	*(6.4)*	*(7.4)*	*(5.6)*	*(5.5)*	*(5.5)*	*(5.4)*	*(5.7)*	*(5.7)*	*(4.7)*	*(4.5)*
**Dementia**	1.7	2.6	2.9	2.9	3.5	2.5	2.6	2.4	2.4	2.5	2.8	2.0	2.1
		*(2.6)*	*(3.0)*	*(2.9)*	*(3.7)*	*(2.5)*	*(2.7)*	*(2.4)*	*(2.5)*	*(2.6)*	*(3.0)*	*(2.0)*	*(2.2)*
**Depression**	10.3	10.6	9.5	11.3	11.1	10.3	8.8	10.5	9.1	10.1	8.2	9.1	7.3
		*(11.0)*	*(10.7)*	*(11.7)*	*(12.4)*	*(10.7)*	*(9.9)*	*(10.8)*	*(10.2)*	*(10.4)*	*(9.2)*	*(9.4)*	*(8.1)*
**Diabetes**	9.2	10.1	9.5	10.7	11.0	9.7	8.5	9.9	8.9	10.0	9.2	8.7	7.3
		*(10.3)*	*(10.4)*	*(11.0)*	*(12.0)*	*(9.9)*	*(9.4)*	*(10.1)*	*(9.6)*	*(10.2)*	*(10.1)*	*(8.9)*	*(7.9)*
**Dorsal pain**	23.5	29.4	27.4	30.7	30.3	28.6	25.7	29.0	26.6	28.5	25.1	26.1	22.7
		*(30.7)*	*(31.1)*	*(32.1)*	*(34.5)*	*(29.9)*	*(29.3)*	*(30.3)*	*(30.0)*	*(29.7)*	*(28.5)*	*(27.1)*	*(25.5)*
**HF**	3.7	5.6	5.6	6.1	6.8	5.3	5.1	5.2	4.9	5.4	5.3	4.3	4.2
		*(5.6)*	*(6.0)*	*(6.2)*	*(7.2)*	*(5.4)*	*(5.4)*	*(5.3)*	*(5.2)*	*(5.5)*	*(5.6)*	*(4.4)*	*(4.4)*

Source: Own depiction

Abbreviations: *CA* pulmonary, bronchial and tracheal cancer, *CHD* coronary heart disease, *COPD* chronic obstructive pulmonary disease, *CVD* cerebrovascular diseases, *HF* heart failure

All in all, our calculations show that all of the ten diseases are expected to increase up until 2060: Diseases of the musculoskeletal system like dorsal pain and arthrosis will be responsible for the majority of the future disease burden within the German population, possibly affecting about 25–27 and 13–15 million people, respectively, by 2060. Diabetes, which is closely related to other diseases like CHD, is expected to impact at least 9.5 million patients in case of expanding morbidity. With up to 7.4 million people affected in 2060, CHD will continue to be the most common cardiovascular disease. The high growth rates of primarily age-related diseases such as CVD or HF are also steep in absolute terms. Only if prevention strategies are successful, the significant increase in number of patients could be alleviated in the long run.

**Fig. 2:**
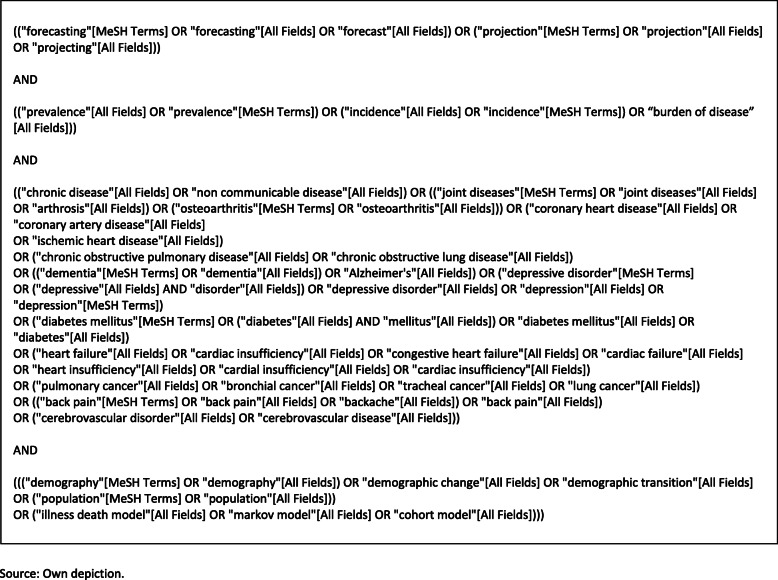
Search filter PubMed

**Fig. 3:**
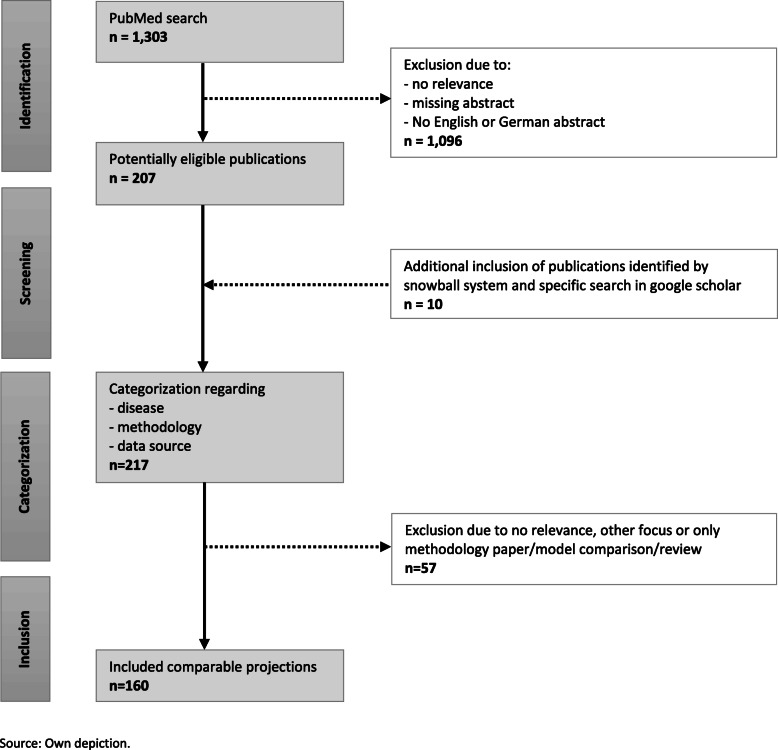
Flow chart of the literature selection process

Our results can be compared with other recent studies for Germany. From the 16 (16/160) studies for Germany in our literature review (concerning our ten most common non-infectious diseases) only six (6/16) were published in the last 5 years and most of them focussing on cancer (3/6), dementia (2/6) or diabetes (1/6)^14^. For diabetes, Tönnies et al. (2019) [29] calculate with the help of an illness-death model and under the assumption of constant incidence rates a higher number of 11.0 million patients for 2040. The discrepancy to our projection (10.3 million) for 2040 is probably due to their older input data, which stem from 2010. The most recent study on dementia by Alzheimer Europe (2020) [30] project 2.7 million patients for 2050 with a status quo projection which lies in the interval of our forecast with 2.5 to 3.0 million people affected. Milan & Fetzer (2019) [17] project 2.6 to 3.3 dementia patients for 2060 by using the same model. The slight differences to their results are attributable to more recent population statistics and disease-specific input data. A comparison of our results with the three studies focusing on cancer is difficult as two of them consider the disease pattern of lung cancer and take a short-term perspective (up to the year 2020), whereas the third focuses on a trend projection of incidence rates (see Fig. 2, Fig. 3 and Table 6 in the appendix for more detailed information and results on the systematic database search).

## Discussion

A projection of ten common non-infectious diseases in concurrent scenarios based on a rich and consistent data set is expanding the literature on the developmentof future disease burden in light of the demographic transition. In this context, ours is one of the few studies using an illness-death approach with recovery and modelling *compression of morbidity *and *prevention *scenarios. Furthermore, due to its time-discrete specification, our model could be directly linked to any (official) population projection, and therefore adapted by institutions in the field of policy consulting.

In contrast to a naïve extrapolation (status quo principle), our analysis highlights the importance of focusing on the interdependence between demographic and disease-specific components in projecting future disease burden. Based on six different scenarios we show the possible future range of disease burden and reveal the large differences between the various diseases in interaction with the demographic components. Considering these differences, it becomes clear that the extrapolation of prevalence rates can only reflect the cohort effect caused by population structure and not epidemiologically induced changes in the burden of disease, as observed e.g. for dorsal pain. In contrast, for CHD the status quo projection ranges, as expected, between the compression and expansion scenarios due to minor epidemiological influences.

With regard to the probability of the different hypotheses on future disease burden, the study situation remains inconclusive. Chatterji et al. (2015) [31] show with their detailed review of studies across the world how much the results vary for observed compression or expansion in recent years. However, just looking on the prevalence of chronic diseases (not e.g. in the quality of life) resulted more frequently in an expansion. Considering very similar diseases as our study in connection with proximity to death, Beltrán-Sánchez et al. (2016) [32] show for the United States that those who died in recent times had a higher prevalence of chronic diseases in periods far from death, especially of those chronic diseases with low mortality and high frequency.

Interestingly, even in international studies there are only a few projections for the two major common diseases dorsal pain and arthrosis (1/160 dorsal pain, 10/160 arthrosis or joint replacement procedures), although these diseases are expected to increase the most in total numbers of patients according to our calculations. Our results can be compared with those of Kingston et al. (2018) [33], who use a population sample to model multimorbidity and prevalence of similar diseases for over 65-year-olds in England until 2035. In line with our findings, they predict a significant increase for all diseases considered except depression, but with the largest increases for cancer, diabetes and respiratory diseases. In line with our findings, the only study that also compares different compression scenarios, but with regard to disability due to similar diseases in the UK, by Jagger et al. (2006) [9], concludes that improvements in population health cannot fully compensate the effect of population ageing and that there will still be an increase in number of older people with disabilities.

Of course, our results are also subject to limitations. The Markov assumption of the illness-death model implies that the transition probabilities depend only on the current state and are not influenced by past events. But complex long-term studies, e.g. on the probability of re-disease after a successful recovery, would be necessary to heal this caveat, which are not available for such a large number of insureds. However, regarding the fit with observed incidence or prevalence rates, multistate models used in a retrospective analysis of epidemiological study data (in contrast to regression models) score well [34, 35].

Even if our discrete model has certain advantages, modelling in discrete time might be overestimating epidemiological effects. By comparing the results of a discrete-time model with those of a continuous model, Brinks & Landwehr (2014) [36] show that a projection in discrete time can overestimate future prevalence. However, the authors also state that smaller projection intervals lead to smaller deviations. Our chosen one-year interval leads to about a 10% overestimation in their model.

Nonetheless, this overestimation effect might be somehow offset by the conservative estimates generated by using insurance data, which constitutes another limitation of our measure. Insurance or routine data is primarily collected for invoicing medical services when patients visit a physician. Thus, the resulting prevalence and incidence rates can only be interpreted as treatment rates and are usually slightly lower than those obtained by surveys. In conjunction with the required validation procedures, the actual population incidence could be underestimated. Due to the incomplete coding observed for some diseases, it is also questionable whether the documented onset of illness corresponds to the real date of incidence.

A third limitation could be our data set: The rates determined from the AOK Baden-Württemberg might differ from the rates of the total German population. However, regarding gender-specific differences or frequencies in older cohorts that are particularly relevant for this analysis, various studies indicate that large AOK data sets are representative [37–39].

Further insights could be obtained by including multi-morbidity in our model^15^. Comorbidity analyses could also provide more detailed insights into causes of mortality differences, which would help limiting the range of possible future scenarios. Despite the limitations mentioned, our results can offer an important guide to rational decisions in health care, especially due to the actuality and detail level of the data used. Although the strongly age-related diseases such as dementia or heart failure show the highest relative increase rates, the enormous prevalence of musculoskeletal diseases and depression should not be ignored. Most importantly, for almost all considered diseases a significant increase in burden of disease can be expected even in case of a compression of morbidity.

## Conclusion

We think that our approach is useful for consulting health care professionals and politicians in preparing for the upcoming pressure on health care capacities. As the current COVID-19 crisis is showing, health care capacities are quite scarce. Even in our most optimistic scenario we would have the same pressure – at least in numbers – from chronic diseases as currently experienced during the pandemic. The lesson from our analysis is clear: A massive case-load is emerging on the German health care system, which can only be alleviated by more effective prevention. Immediate action by policy makers and health care managers is needed, as otherwise the prevalence of widespread diseases will become unsustainable from a capacity point-of-view.

## Data Availability

Population data is available at Destatis, Germany’s official statistical office, and mortality.org. The aggregate claim data from the German sickness fund is available upon request depending on the permission of the data donor.

## Appendix

**Table 6:** Bibliography and study characteristics

#	Study	Country	Disease	Data source	Methodology	Projected year	DOI/PMID	Source
1	Cho et al. (2018): “IDF Diabetes Atlas: Global estimates of diabetes prevalence for 2017 and projections for 2045”	Global	Diabetes	Literature review	Trend extrapolation (Status quo)	2045	10.1016/j.diabres.2018.02.023	Diabetes Research and Clinical Practice
2	Hebert et al. (2013): “Alzheimer disease in the United States (2010–2050) estimated using the 2010 census”	USA	Dementia/ Alzheimer’s	Other epidemiological studies	Multistate model	2050	10.1212/WNL.0b013e31828726f5	Neurology
3	Shaw et al. (2010): “Global estimates of the prevalence of diabetes for 2010 and 2030”	Global	Diabetes	Literature review	Trend extrapolation (Status quo)	2030	10.1016/j.diabres.2009.10.007	Diabetes Research and Clinical Practice
4	Guariguata et al. (2014): “Global estimates of diabetes prevalence for 2013 and projections for 2035”	Global	Diabetes	Literature review	Trend extrapolation (Status quo)	2035	10.1016/j.diabres.2013.11.002. Epub 2013 Dec 1.	Diabetes Research and Clinical Practice
5	Ferri et al. (2005): “Global prevalence of dementia: a Delphi consensus study”	Global	Dementia/ Alzheimer’s	Literature review	Trend extrapolation (Status quo)	2040	10.1016/S0140-6736(05)67889-0	The Lancet
6	Rowley et al. (2017): “Diabetes 2030: Insights from Yesterday, Today, and Future Trends”	USA	Diabetes	Literature review	Multistate model	2030	10.1089/pop.2015.0181	Population Health Management
7	Meza et al. (2015): “Burden of type 2 diabetes in Mexico: past, current and future prevalence and incidence rates”	Mexico	Diabetes	Survey	Multistate model and trend extrapolation	2050	10.1016/j.ypmed.2015.10.015	Preventive Medicine
8	Brookmeyer et al. (2018): “Forecasting the prevalence of preclinical and clinical Alzheimer’s disease in the United States”	USA	Dementia/ Alzheimer’s	Other epidemiological studies	Multistate model	2060	10.1016/j.jalz.2017.10.009	Alzheimers and Dementia
9	Etkind et al. (2017): “How many people will need palliative care in 2040? Past trends, future projections and implications for services”	GB	Sundries	Mixed data sources	Trend extrapolation (Status quo)	2040	10.1186/s12916-017-0860-2	BMC Medicine
10	Bommer et al. (2018) “Global Economic Burden of Diabetes in Adults: Projections From 2015 to 2030”	Global	Diabetes	Literature review	Trend extrapolation (Status quo)	2030	10.2337/dc17-1962	Diabetes Care
11	Wong et al. (2017): “Projection of prediabetes and diabetes population size in Singapore using a dynamic Markov model”	Singapore	Diabetes	Registries	Multistate model	2035	10.1111/1753-0407.12384	Journal of Diabetes
12	Morrell et al. (2016): “Diabetes incidence and projections from prevalence surveys in Fiji”	Fiji	Diabetes	Survey	Trend extrapolation	2020	10.1186/s12963-016-0114-0	Population Health Metrics
13	Kingston et al. (2016): “Projections of multi-morbidity in the older population in England to 2035: estimates from the Population Ageing and Care Simulation (PACSim) model”	GB	Various	Survey	Multistate model	2040	10.1093/ageing/afx201	Age and Ageing
14	Brookmeyer et al. (1998): “Projections of Alzheimer’s disease in the United States and the public health impact of delaying disease onset”	USA	Dementia/ Alzheimer’s	Literature review	Multistate model (Illness-death model)	2050	10.2105/ajph.88.9.1337	American Journal of Public Health
15	Gonzales-Gonzales et al. (2017): “Projecting diabetes prevalence among Mexicans aged 50 years and older: the Future Elderly Model-Mexico (FEM-Mexico)”	Mexico	Diabetes	Survey	Trend extrapolation	2050	10.1136/bmjopen-2017-017330	BMJ Open
16	Ziegler-Graham et al. (2008): “Estimating the prevalence of limb loss in the United States: 2005 to 2050”	USA	Others	Routine data	Multistate model (Illness-death model)	2050	10.1016/j.apmr.2007.11.005	Archives of Physical Medicine and Rehabilitation
17	Lin et al. (2018): “Projection of the future diabetes burden in the United States through 2060”	USA	Diabetes	Survey	Multistate model (Illness-death model)	2060	10.1186/s12963-018-0166-4	Population Health Metrics
18	Matthews et al. (2019): “Racial and ethnic estimates of Alzheimer’s disease and related dementias in the United States (2015–2060) in adults aged ≥65 years”	USA	Dementia/ Alzheimer’s	Routine data	Trend extrapolation (Status quo)	2060	10.1016/j.jalz.2018.06.3063	Alzheimers & Dementia
19	Awad et al. (2018): “Forecasting the burden of type 2 diabetes mellitus in Qatar to 2050: A novel modeling approach”	Qatar	Diabetes	Survey	Multistate model	2050	10.1016/j.diabres.2017.11.015	Diabetes Research and Clinical Practice
20	Meo (2016): “Prevalence and future prediction of type 2 diabetes mellitus in the Kingdom of Saudi Arabia: A systematic review of published studies”	Saudi Arabia	Diabetes	Literature review	Trend extrapolation (Status quo)	2030	PMID: 27339576	Journal of the Pakistan Medical Association
21	Hebert et al. (2003): “Alzheimer disease in the US population: prevalence estimates using the 2000 census”	USA	Dementia/ Alzheimer’s	Other epidemiological studies	Multistate model	2050	10.1001/archneur.60.8.1119	Archives of Neurology
22	Savica et al. (2018): “Parkinson disease with and without Dementia: A prevalence study and future projections”	USA	Others	Other epidemiological studies	Trend extrapolation (Status quo)	2060	10.1002/mds.27277	Movement Disorders
23	Ackerman et al. (2018): “Projected Burden of Osteoarthritis and Rheumatoid Arthritis in Australia: A Population-Level Analysis”	Australia	Various	Survey	Trend extrapolation (Status quo)	2030	10.1002/acr.23414	Arthritis Care & Research
24	Ahmadi-Abhari et al. (2017); “Temporal trend in dementia incidence since 2002 and projections for prevalence in England and Wales to 2040: modelling study”	GB	Dementia/ Alzheimer’s	Survey	Multistate model	2040	10.1136/bmj.j2856.	British Medical Journal
25	Sugiyama et al. (2017): “Construction of a simulation model and evaluation of the effect of potential interventions on the incidence of diabetes and initiation of dialysis due to diabetic nephropathy in Japan”	Japan	Diabetes	Survey	Trend extrapolation	2035	10.1186/s12913-017-2784-0	BMC Health Services Research
26	Sarink et al. (2016): “Projected age- and sex-specific prevalence of cardiovascular diseases in Western Australian adults from 2005 to 2045”	Australia	Various	Mixed data sources	Multistate model	2045	10.1177/2047487314554865	European Journal of Preventive Cardiology
27	Imperatore et al. (2012): “Projections of type 1 and type 2 diabetes burden in the U.S. population aged < 20 years through 2050: dynamic modeling of incidence, mortality, and population growth”	USA	Diabetes	Other epidemiological studies	Multistate model	2050	10.2337/dc12-0669	Diabetes Care
28	Andersson et al. (2015): “Diabetes Prevalence in Sweden at Present and Projections for Year 2050”	Sweden	Diabetes	Mixed data sources	Multistate model (Illness-death model)	2050	10.1371/journal.pone.0143084	PLoS One
29	Kainz et al. (2015): “Prediction of prevalence of chronic kidney disease in diabetic patients in countries of the European Union up to 2025”	Various	Various	Other epidemiological studies	Trend extrapolation	2025	10.1093/ndt/gfv073.	Nephrology Dialysis Transplantation
30	Manuel et al. (2016): “Alzheimer’s and other dementias in Canada, 2011 to 2031: a microsimulation Population Health Modeling (POHEM) study of projected prevalence, health burden, health services, and caregiving use”	Canada	Dementia/ Alzheimer’s	Routine data	Trend extrapolation	2031	10.1186/s12963-016-0107-z	Population Health Metrics
31	de Sousa-Uva et al. (2016): “Trends in diabetes incidence from 1992 to 2015 and projections for 2024: A Portuguese General Practitioner’s Network study”	Portugal	Diabetes	Other epidemiological studies	Trend extrapolation	2024	10.1016/j.pcd.2016.05.003	Primary Care Diabetes
32	Saidi et al. (2015): “Forecasting Tunisian type 2 diabetes prevalence to 2027: validation of a simple model”	Tunisia	Diabetes	Mixed data sources	Multistate model	2027	10.1186/s12889-015-1416-z	BMC Public Health
33	Olajide et al. (2015): “Lung cancer trend in England for the period of 2002 to 2011 and projections of future burden until 2020”	GB	Pulmonary cancer	Registries	Trend extrapolation	2020	10.3892/ijo.2015.3049	International Journal of Oncology
34	Javanbakht et al. (2015): “Projection of Diabetes Population Size and Associated Economic Burden through 2030 in Iran: Evidence from Micro-Simulation Markov Model and Bayesian Meta-Analysis”	Iran	Diabetes	Survey	Multistate model	2030	10.1371/journal.pone.0132505. eCollection 2015.	PLoS One
35	Turkiewicz et al. (2014): “Current and future impact of osteoarthritis on health care: a population-based study with projections to year 2032”	Sweden	Arthrosis	Routine data	Trend extrapolation (Status quo)	2032	10.1016/j.joca.2014.07.015. Epub 2014 Jul 30.	Osteoarthritis and Cartilage
36	Bilandzic & Rosella (2017): “The cost of diabetes in Canada over 10 years: applying attributable health care costs to a diabetes incidence prediction model”	Canada	Diabetes	Survey	Trend extrapolation	2022	10.24095/hpcdp.37.2.03	Health Promotion and Chronic Disease Prevention in Canada: Research, Policy and Practice
37	Al Ali et al. (2013): “Forecasting future prevalence of type 2 diabetes mellitus in Syria”	Syria	Diabetes	Survey	Multistate model	2022	10.1186/1471-2458-13-507.	BMC Public Health
38	Park et al. (2013): “Burden of disease due to dementia in the elderly population of Korea: present and future”	Korea	Dementia/ Alzheimer’s	Literature review	Trend extrapolation (Status quo)	2050	10.1186/1471-2458-13-293.	BMC Public Health
39	Waldeyer et al. (2013): “Projection of the burden of type 2 diabetes mellitus in Germany: a demographic modelling approach to estimate the direct medical excess costs from 2010 to 2040”	Germany	Diabetes	Survey	Multistate model (Illness-death model)	2040	10.1111/dme.12177	Diabetic Medicine
40	Milan & Fetzer (2019): “The future development of dementia diseases in Germany-a comparison of different forecast models”	Germany	Dementia/ Alzheimer’s	Routine data	Multistate model (Illness-death model)	2060	10.1007/s00103-019-02981-3	Bundesgesund-heitsblatt Gesundheits-forschung Gesundheits-schutz
41	Loef & Walach (2013): “Midlife obesity and dementia: meta-analysis and adjusted forecast of dementia prevalence in the United States and China”	Various	Dementia/ Alzheimer’s	Literature review	Trend extrapolation (Status quo)	2030	10.1002/oby.20037	Obesity
42	Png et al. (2016): “Current and future economic burden of diabetes among working-age adults in Asia: conservative estimates for Singapore from 2010 to 2050”	Singapore	Diabetes	Mixed data sources	Trend extrapolation (Status quo)	2050	10.1186/s12889-016-2827-1.	BMC Public Health
43	Al-Quwaidhi et al. (2014): “Comparison of type 2 diabetes prevalence estimates in Saudi Arabia from a validated Markov model against the International Diabetes Federation and other modelling studies”	Saudi Arabia	Diabetes	Survey	Multistate model	2030	10.1016/j.diabres.2013.12.036	Diabetes Research and Clinical Practice
44	Backholer et al. (2013): “Diabetes Prevention and Treatment Strategies - Are we doing enough?”	Australia	Diabetes	Other epidemiological studies	Multistate model	2025	10.2337/dc12-2501	Diabetes Care
45	Huang et al. (2009): “Projecting the Future Diabetes Population Size and Related Costs for the U.S.”	USA	Diabetes	Survey	Multistate model	2033	10.2337/dc09-0459	Diabetes Care
46	Tobias et al. (2008): “Burden of Alzheimer’s disease: population-based estimates and projections for New Zealand, 2006–2031”	New Zealand	Dementia/ Alzheimer’s	Literature review	Multistate model	2031	10.1080/00048670802277297.	Australian and New Zealand Journal of Psychiatry
47	Dall et al. (2013): “An aging population and growing disease burden will require a large and specialized health care workforce by 2025”	USA	Various	Survey	Trend extrapolation	2025	10.1377/hlthaff.2013.0714.	Health Affairs
48	Boyle et al. (2001): “Projection of diabetes burden through 2050: impact of changing demography and disease prevalence in the U.S.”	USA	Diabetes	Survey	Trend extrapolation	2050	10.2337/diacare.24.11.1936	Diabetes Care
49	Burgel et al. (2018): “An attempt at modeling COPD epidemiological trends in France”	France	COPD	Mixed data sources	Multistate model	2025	10.1186/s12931-018-0827-7.	Respiratory Research
50	Jorm et al. (2005): “Projections of future numbers of dementia cases in Australia with and without prevention”	Australia	Dementia/ Alzheimer’s	Other epidemiological studies	Trend extrapolation (Status quo)	2050	10.1080/j.1440-1614.2005.01713.x	Australian and New Zealand Journal of Psychiatry
51	Sloane et al. (2002): “The public health impact of Alzheimer’s disease, 2000–2050: potential implication of treatment advances”	USA	Dementia/ Alzheimer’s	Literature review	Multistate model	2050	10.1146/annurev.publhealth.23.100901.140525	Annual Review of Public Health
52	Heo et al. (2008): “Population projection of US adults with lifetime experience of depressive disorder by age and sex from year 2005 to 2050”	USA	Depression	Survey	Trend extrapolation (Status quo)	2050	10.1002/gps.2061	International Journal of Geriatric Psychiatry
53	Brookmeyer & Gray (2000): “Methods for projecting the incidence and prevalence of chronic diseases in aging populations: application to Alzheimer’s disease”	USA	Dementia/ Alzheimer’s	Literature review	Multistate model (Illness-death model)	2050	10.1002/(sici)1097-0258(20,000,615/30)19:11/12 < 1481::aid-sim440 > 3.0.co;2-u	Statistics in Medicine
54	Centers for Disease Control and Prevention (CDC) (2003): “Public health and aging: projected prevalence of self-reported arthritis or chronic joint symptoms among persons aged > 65 years--United States, 2005–2030”	USA	Arthritis	Survey	Trend extrapolation (Status quo)	2030	PMID: 12809109	MMWR Morbidity and Mortality Weekly Report
55	Odden et al. (2011): “The Impact of the Aging Population on Coronary Heart Disease in the U.S.”	USA	CHD	Mixed data sources	Multistate model	2040	10.1016/j.amjmed.2011.04.010	American Journal of Medicine
56	Brinks et al. (2012): “Prevalence of type 2 diabetes in Germany in 2040: estimates from an epidemiological model”	Germany	Diabetes	Survey	Multistate model (Illness-death model)	2040	10.1007/s10654-012-9726-2.	European Journal of Epidemiology
57	Stewart et al. (2003): “Heart failure and the aging population: an increasing burden in the twenty-first century?”	GB	Heart failure	Mixed data sources	Trend extrapolation (Status quo)	2020	10.1136/heart.89.1.49	Heart
58	Holt et al. (2011): “Forecasting the burden of advanced knee osteoarthritis over a 10-year period in a cohort of 60–64 year-old US adults”	USA	Arthrosis	Mixed data sources	Multistate model	2020	10.1016/j.joca.2010.10.009	Osteoarthritis and Cartilage
59	Saaddine et al. (2008): “Projection of diabetic retinopathy and other major eye diseases among people with diabetes mellitus: United States, 2005–2050”	USA	Various	Survey	Multistate model(Illness-death model)	2050	10.1001/archopht.126.12.1740.	Archives of Ophthalmology
60	Vickland et al. (2011): “Who pays and who benefits? How different models of shared responsibilities between formal and informal carers influence projections of costs of dementia management”	Australia	Dementia/ Alzheimer’s	Literature review	Trend extrapolation	2040	10.1186/1471-2458-11-793	BMC Public Health
61	Moran et al. (2008): “The future impact of population growth and aging on coronary heart disease in China: projections from the Coronary Heart Disease Policy Model-China”	China	CHD	Mixed data sources	Multistate model	2030	10.1186/1471-2458-8-394	BMC Public Health
62	Jagger et al. (2009): “The effect of dementia trends and treatments on longevity and disability: a simulation model based on the MRC Cognitive Function and Ageing Study (MRC CFAS)”	GB	Dementia/ Alzheimer’s	Other epidemiological studies	Multistate model	2026	10.1093/ageing/afp016	Age and Ageing
63	Ackerman et al. (2019): “The projected burden of primary total knee and hip replacement for osteoarthritis in Australia to the year 2030”	Australia	Joint replacement	Registries	Trend extrapolation (Status quo)	2030	10.1186/s12891-019-2411-9	BMC Musculoskeletal Disorders
64	Schaubel et al. (1995): “End-stage renal disease projections for Canada to 2005 using Poisson and Markov models”	Canada	Kidney disease	Registries	Multistate model and trend extrapolation	2005	10.1093/ije/27.2.274	International Journal of Epidemiology
65	Mura et al. (2010): “How many dementia cases in France and Europe? Alternative projections and scenarios 2010–2050”	Various	Dementia/ Alzheimer’s	Other epidemiological studies	Multistate model	2050	10.1111/j.1468-1331.2009.02783.x	European Journal of Neurology
66	Parsons & Somervaille (2000): “Estimation and projection of population lung cancer trends (United Kingdom)”	GB	Pulmonary cancer	Registries	Trend extrapolation	2015	10.1023/a:1008966125578	Cancer Causes & Control
67	Soerjomataram et al. (2011): “Reducing inequalities in lung cancer incidence through smoking policies”	Netherlands	Pulmonary cancer	Survey	Trend extrapolation	2050	10.1016/j.lungcan.2011.01.009	Lung Cancer
68	Robertsson et al. (2000): “Past incidence and future demand for knee arthroplasty in Sweden: a report from the Swedish Knee Arthroplasty Register regarding the effect of past and future population changes on the number of arthroplasties performed”	Sweden	Joint replacement	Registries	Trend extrapolation (Status quo)	2030	10.1080/000164700317393376	Acta Orthopaedica Scandinavica
69	Murakami & Ohashi (2001): “Projected number of diabetic renal disease patients among insulin-dependent diabetes mellitus children in Japan using a Markov model with probabilistic sensitivity analysis”	Japan	Kidney disease	Literature review	Multistate model	2015	10.1093/ije/30.5.1078	International Journal of Epidemiology
70	Pritzkuleit et al. (2010): “Disease numbers in pneumology - a projection to 2060”	Germany	Various	Literature review	Trend extrapolation (Status quo)	2060	10.1055/s-0030-1,255,637	Pneumologie
71	Rowley & Bezold (2012): “Creating public awareness: state 2025 diabetes forecasts”	USA	Diabetes	Literature review	Multistate model	2025	10.1089/pop.2011.0053	Population Health Management
72	Narayan et al. (2006): “Impact of recent increase in incidence on future diabetes burden: U.S., 2005–2050”	USA	Diabetes	Survey	Multistate model (Illness-death model)	2006	10.2337/dc06-1136	Diabetes Care
73	Fontaine et al. (2007): “Projected prevalence of US adults with self-reported doctor-diagnosed arthritis, 2005 to 2050”	USA	Arthritis	Survey	Trend extrapolation (Status quo)	2050	10.1007/s10067-007-0556-7	Clinical Rheumatology
74	Ruwaard et al. (1993): “Forecasting the number of diabetic patients in The Netherlands in 2005”	Netherlands	Diabetes	Mixed data sources	Multistate model (Illness-death model)	2005	10.2105/ajph.83.7.989	American Journal of Public Health
75	Hebert et al. (2004): “State-specific projections through 2025 of Alzheimer disease prevalence”	USA	Dementia/Alzheimer’s	Other epidemiological studies	Trend extrapolation (Status quo)	2025	10.1212/01.wnl.0000123018.01306.10	Neurology
76	Gao et al. (2017): “Nephrology Dialysis Transplantation”	Canada	Various	Mixed data sources	Trend extrapolation	2025	10.1186/s12882-017-0699-y	BMC Nephrology
77	Feenstra et al. (2001): “The impact of aging and smoking on the future burden of chronic obstructive pulmonary disease: a model analysis in the Netherlands”	Netherlands	COPD	Other epidemiological studies	Multistate model	2015	10.1164/ajrccm.164.4.2003167	American Journal of Respiratory and Critical Care Medicine
78	Danielsen et al. (2017): “Prevalence of heart failure in the elderly and future projections: the AGES-Reykjavík study”	Iceland	Heart failure	Other epidemiological studies	Trend extrapolation (Status quo)	2060	10.1080/14017431.2017.1311023	Scandinavian Cardiovascular Journal
79	Sharif et al. (2015): “Projecting the direct cost burden of osteoarthritis in Canada using a microsimulation model”	Canada	Arthrosis	Mixed data sources	Trend extrapolation	2031	10.1016/j.joca.2015.05.029	Osteoarthritis and Cartilage
80	Gouveia et al. (2019): “The current and future burden of heart failure in Portugal”	Portugal	Heart failure	Mixed data sources	Trend extrapolation (Status quo)	2036	10.1002/ehf2.12399	ESC Heart Failure
81	Standfield et al. (2018): “A simulation of dementia epidemiology and resource use in Australia”	Australia	Dementia/ Alzheimer’s	Literature review	Trend extrapolation	2050	10.1111/1753-6405.12700	Australian and New Zealand Journal of Public Health
82	Menvielle et al. (2010): “Scenarios of future lung cancer incidence by educational level: Modelling study in Denmark”	Denmark	Pulmonary cancer	Mixed data sources	Trend extrapolation	2050	10.1016/j.ejca.2010.07.027	European Journal of Cancer
83	Peters et al. (2010): “Demographic change and disease rates: a projection until 2050”	Germany	Various	Literature review	Trend extrapolation (Status quo)	2050	10.1007/s00103-010-1050-y	Bundesgesund-heitsblatt Gesundheits-forschung Gesundheits-schutz
84	Brinks et al. (2014): “Age-specific prevalence of diagnosed systemic lupus erythematosus in Germany 2002 and projection to 2030”	Germany	Others	Routine data	Trend extrapolation (Status quo)	2030	10.1177/0961203314540352	Lupus
85	Oberaigner & Geiger-Gritsch (2014): “Prediction of cancer incidence in Tyrol/Austria for year of diagnosis 2020”	Austria	Cancer	Registries	Trend extrapolation	2020	10.1007/s00508-014-0596-3	Wiener klinische Wochenschrift
86	Nowossadeck et al.: (2014): “The future incidence of colorectal and lung cancers: results of the calculation of different scenarios for the year 2020”	Germany	Cancer	Registries	Trend extrapolation (Status quo)	2020	10.1007/s00103-013-1873-4	Bundesgesund-heitsblatt Gesundheits-forschung Gesundheits-schutz
87	Bonneux et al. (1994): “Estimating clinical morbidity due to ischemic heart disease and congestive heart failure: the future rise of heart failure”	Netherlands	Heart failure	Mixed data sources	Multistate model	2010	10.2105/ajph.84.1.20	American Journal of Public Health
88	Nowatzki et al. (2011): “Projection of future cancer incidence and new cancer cases in Manitoba, 2006–2025”	Canada	Cancer	Registries	Trend extrapolation	2025	PMID: 21466757	Chronic Diseases in Canada
89	Salomaa et al. (2013): “Aging of the population may not lead to an increase in the numbers of acute coronary events: a community surveillance study and modelled forecast of the future”	Finland	CHD	Registries	Trend extrapolation	2050	10.1136/heartjnl-2012-303,216	Heart
90	Didkowska et al. (2011): “Future lung cancer incidence in Poland and Finland based on forecasts on hypothetical changes in smoking habits”	Various	Pulmonary cancer	Mixed data sources	Trend extrapolation	2030	10.3109/0284186X.2010.488247	Acta Oncologica
91	Estes et al. (2018): “Modeling NAFLD disease burden in China, France, Germany, Italy, Japan, Spain, United Kingdom, and United States for the period 2016–2030”	Various	Others	Literature review	Multistate model	2030	10.1016/j.jhep.2018.05.036	Journal of Hepatology
92	McLean et al. (2016): “Projecting the COPD population and costs in England and Scotland: 2011 to 2030”	GB	COPD	Mixed data sources	Multistate model	2030	10.1038/srep31893	Scientific Reports
93	Firlei et al. (2007): “The prevalence of COPD in Austria--the expected change over the next decade”	Austria	COPD	Other epidemiological studies	Trend extrapolation (Status quo)	2020	10.1007/s00508-007-0867-3	Wiener klinische Wochenschrift
94	Cobiac & Scarborough (2017): “Translating the WHO 25 × 25 goals into a UK context: the PROMISE modelling study”	GB	Various	Mixed data sources	Trend extrapolation	2030	10.1136/bmjopen-2016-012805	BMJ Open
95	Marimuthu (2008): “Projection of cancer incidence in five cities and cancer mortality in India”	India	Cancer	Registries	Trend extrapolation (Status quo)	2008	PMID: 18453733	Indian Journal of Cancer
96	Soerjomataram et al. (2010): “Impact of a smoking and alcohol intervention programme on lung and breast cancer incidence in Denmark: An example of dynamic modelling with Prevent”	Denmark	Pulmonary cancer	Mixed data sources	Trend extrapolation	2050	10.1016/j.ejca.2010.07.051	European Journal of Cancer
97	Edwards et al. (2014): “A novel approach for the accurate prediction of thoracic surgery workforce requirements in Canada”	Canada	Pulmonary cancer	Survey	Trend extrapolation	2030	10.1016/j.jtcvs.2014.03.031	The Journal of Thoracic and Cardiovascular Surgery
98	Estes et al. (2018): “Modeling the epidemic of nonalcoholic fatty liver disease demonstrates an exponential increase in burden of disease”	USA	Others	Literature review	Multistate model	2030	10.1002/hep.29466	Hepatology
99	Perruccio et al. (2006): “Revisiting arthritis prevalence projections--it’s more than just the aging of the population”	Canada	Arthritis	Survey	Trend extrapolation	2021	PMID: 16960946	Journal of Rheumatology
100	Nepal et al. (2014): “Rising midlife obesity will worsen future prevalence of dementia”	Australia	Dementia/ Alzheimer’s	Literature review	Trend extrapolation (Status quo)	2050	10.1371/journal.pone.0099305	PLoS One
101	Rahib et al. (2014): “Projecting cancer incidence and deaths to 2030: the unexpected burden of thyroid, liver, and pancreas cancers in the United States”	USA	Cancer	Mixed data sources	Trend extrapolation (Status quo)	2030	10.1158/0008-5472.CAN-14-0155	Cancer Research
102	Orenstein & Shi (2016): “Microsimulation Modeling of Coronary Heart Disease: Maximizing the Impact of Nonprofit Hospital-Based Interventions”	USA	CHD	Survey	Trend extrapolation	2030	10.1177/0046958016666009	INQUIRY: The Journal of Health Care Organization, Provision, and Financing
103	Quante et al. (2016): “Projections of cancer incidence and cancer-related deaths in Germany by 2020 and 2030”	Germany	Cancer	Registries	Trend extrapolation	2030	10.1002/cam4.767	Cancer Medicine
104	Gilbertson et al. (2005): “Projecting the number of patients with end-stage renal disease in the United States to the year 2015”	USA	Kidney disease	Survey	Multistate model	2005	10.1681/ASN.2005010112	Journal of the American Society of Nephrology
105	Beelte et al. (2008): “Lung cancer incidence and mortality: current trends and projections based on data from Schleswig-Holstein”	Germany	Pulmonary cancer	Registries	Trend extrapolation (Status quo)	2020	10.1055/s-2008-1,081,095	Deutsche Medizinische Wochenschrift
106	Tsoi et al. (2017): “Cancer burden with ageing population in urban regions in China: projection on cancer registry data from World Health Organization”	China	Cancer	Registries	Trend extrapolation	2030	10.1093/bmb/ldw050	British Medical Bulletin
107	Okura et al. (2008): “Impending epidemic: future projection of heart failure in Japan to the year 2055”	Japan	Heart failure	Other epidemiological studies	Trend extrapolation (Status quo)	2055	10.1253/circj.72.489	Circulation Journal
108	Ansah et al. (2018): “Projection of Eye Disease Burden in Singapore”	Singapore	Others	Other epidemiological studies	Trend extrapolation (Status quo)	2040	PMID: 29493707	Annals Academy of Medicine Singapore
109	Heidenreich et al. (2011): “Forecasting the future of cardiovascular disease in the United States: a policy statement from the American Heart Association”	USA	Various	Survey	Trend extrapolation (Status quo)	2030	10.1161/CIR.0b013e31820a55f5	Circulation
110	Bahr et al. (2015): “Prognosis of population-related morbidity for common cancers in Germany--Effects on health care”	Germany	Cancer	Registries	Trend extrapolation (Status quo)	2020	10.1055/s-0041-101,356	Deutsche Medizinische Wochenschrift
111	Weinstein et al. (1987): “Forecasting coronary heart disease incidence, mortality, and cost: the Coronary Heart Disease Policy Model”	USA	CHD	Mixed data sources	Multistate model	2010	10.2105/ajph.77.11.1417	American Journal of Public Health
112	Campbell et al. (2018): “The present and future burden of previously treated advanced non-small cell lung cancer (NSCLC) by histology and line of therapy in France, Germany, Italy, and Spain: model-based predictions”	Various	Pulmonary cancer	Registries	Trend extrapolation	2020	10.1186/s12963-018-0174-4	Population Health Metrics
113	Moran et al. (2010): “Future cardiovascular disease in china: markov model and risk factor scenario projections from the coronary heart disease policy model-china”	China	CHD	Other epidemiological studies	Multistate model and trend extrapolation	2020	10.1161/CIRCOUTCOMES.109.910711	Circulation: Cardiovascular Quality and Outcomes
114	Kingston et al. (2018): “Forecasting the care needs of the older population in England over the next 20 years: estimates from the Population Ageing and Care Simulation (PACSim) modelling study”	GB	Various	Other epidemiological studies	Multistate model	2035	10.1016/S2468-2667(18)30118-X	The Lancet Public Health
115	Baik (2019): “Projection of Diabetes Prevalence in Korean Adults for the Year 2030 Using Risk Factors Identified from National Data”	Korea	Diabetes	Survey	Trend extrapolation	2030	10.4093/dmj.2018.0043	Diabetes and Metabolism Journal
116	Lee et al. (2016): “Epidemiology of Heart Failure in Korea: Present and Future”	Korea	Heart failure	Routine data	Trend extrapolation (Status quo)	2040	10.4070/kcj.2016.46.5.658	Korean Circulation Journal
117	Mukasheva et al. (2019): “Forecasting the Prevalence of Diabetes Mellitus Using Econometric Models”	Kazakhstan	Diabetes	Registries	Trend extrapolation	2018	10.1007/s13300-019-00684-1	Diabetes Therapy
118	Pandya et al. (2013): “More americans living longer with cardiovascular disease will increase costs while lowering quality of life”	USA	CHD	Survey	Trend extrapolation	2030	10.1377/hlthaff.2013.0449	Health Affairs
119	Pan et al. (2010): “Burden of diabetes in the adult Chinese population: A systematic literature review and future projections”	China	Diabetes	Literature review	Trend extrapolation (Status quo)	2016	10.2147/ijgm.s6343	International Journal of General Medicine
120	Chen et al. (2011): “Bayesian age-period-cohort prediction of lung cancer incidence in China”	China	Pulmonary cancer	Registries	Trend extrapolation	2020	10.1111/j.1759-7714.2011.00062.x	Thoracic Cancer
121	Brookmeyer et al. (2007): “Forecasting the global burden of Alzheimer’s disease”	Global	Dementia/ Alzheimer’s	Literature review	Multistate model	2050	10.1016/j.jalz.2007.04.381	Alzheimers and Dementia
122	Boyle et al. (2010): “Projection of the year 2050 burden of diabetes in the US adult population: dynamic modeling of incidence, mortality, and prediabetes prevalence”	USA	Diabetes	Literature review	Multistate model and trend extrapolation	2050	10.1186/1478-7954-8-29	Population Health Metrics
123	Wong et al. (2018): “Projecting the Burden of Chronic Kidney Disease in a Developed Country and Its Implications on Public Health”	Singapore	Kidney disease	Registries	Multistate model	2035	10.1155/2018/5196285	International Journal of Nephrology
124	Bai et al. (2018): “The trends and projections in the incidence and mortality of liver cancer in urban Shanghai: a population-based study from 1973 to 2020”	Shanghai	Cancer	Registries	Trend extrapolation	2020	10.2147/CLEP.S153951	Clinical Epidemiology
125	Islek et al. (2016): “Estimating the potential contribution of stroke treatments and preventative policies to reduce the stroke and ischemic heart disease mortality in Turkey up to 2032: a modelling study”	Turkey	CHD	Mixed data sources	Multistate model	2032	10.1186/s12889-015-2655-8	BMC Public Health
126	Guzman-Castillo et al. (2017): “Forecasted trends in disability and life expectancy in England and Wales up to 2025: a modelling study”	GB	Various	Other epidemiological studies	Multistate model	2025	10.1016/S2468-2667(17)30091-9	The Lancet Public Health
127	Phan et al. (2014): “Forecasting the burden of type 2 diabetes in Singapore using a demographic epidemiological model of Singapore”	Singapore	Diabetes	Mixed data sources	Multistate model	2050	10.1136/bmjdrc-2013-000012	BMJ Open Diabetes Research and Care
128	Suka et al. (2004): “The national burdens of rheumatoid arthritis and osteoarthritis in Japan: projections to the year 2010, with future changes in severity distribution”	Japan	Various	Survey	Trend extrapolation (Status quo)	2010	10.3109/s10165-004-0310-9	Modern Rheumatology
129	Earnest et al. (2019): “Forecasting annual incidence and mortality rate for prostate cancer in Australia until 2022 using autoregressive integrated moving average (ARIMA) models”	Australia	Cancer	Registries	Trend extrapolation	2022	10.1136/bmjopen-2019-031331	BMJ Open
130	Wancata et al. (2003): “Number of dementia sufferers in Europe between the years 2000 and 2050”	Various	Dementia/ Alzheimer’s	Literature review	Trend extrapolation (Status quo)	2050	10.1016/j.eurpsy.2003.03.003	European Psychiatry
131	Li et al. (2019): “Prevalence, incidence and future projection of diabetic eye disease in Europe: a systematic review and meta-analysis”	Various	Others	Literature review	Trend extrapolation (Status quo)	2050	10.1007/s10654-019-00560-z	European Journal of Epidemiology
132	Joly et al. (2013): “Prevalence Projections of Chronic Diseases and Impactof Public Health Intervention”	France	Dementia/ Alzheimer’s	Other epidemiological studies	Multistate model (Illness-death model)	2030	10.1111/j.1541-0420.2012.01827.x	Biometrics
133	Mathers & Loncar (2008): “Projections of Global Mortality and Burden of Disease from 2002 to 2030”	Global	Various	Other epidemiological studies	Trend extrapolation	2030	10.1371/journal.pmed.0030442	PLoS Medicine
134	Jagger et al. (2006): “Compression or expansion of disability?: forecasting future disability levels under changing patterns of diseases”	GB	Various	Mixed data sources	Multistate model	2025	http://eprints.lse.ac.uk/4459/1/Compression_or_expansion_of_disability_forecasting_future_disability_levels_under_changing_patterns_of_diseases.%28LSERO%29.pdf	Kings’s Fund
135	Xie et al. (2015): “Cancer incidence in Canada: trends and projections (1983–2032)”	Canada	Cancer	Registries	Trend extrapolation	2032	PMID: 26011811	Health Promotion and Chronic Disease Prevention in Canada
136	Jacqmin-Gadda et al. (2013): “20-Year prevalence projections for dementia and impact of preventive policy about risk factors”	France	Dementia/ Alzheimer’s	Other epidemiological studies	Multistate model(Illness-death model)	2030	10.1007/s10654-013-9818-7	European Journal of Epidemiology
137	Culliford et al. (2015): “Future projections of total hip and knee arthroplasty in the UK: results from the UK Clinical Practice Research Datalink”	GB	Joint replacement	Mixed data sources	Trend extrapolation (Status quo)	2035	10.1016/j.joca.2014.12.022	Osteoarthritis and Cartilage
138	Van Meijgaard et al. (2011): “Forecasting diabetes prevalence in California: a microsimulation”	USA	Diabetes	Mixed data sources	Trend extrapolation	2020	PMID: 21672404	Preventing Chronic Disease
139	Honeycutt et al. (2003): “A dynamic Markov model for forecasting diabetes prevalence in the United States through 2050”	USA	Diabetes	Survey	Multistate model(Illness-death model)	2050	10.1023/a:1024467522972	Health Care Management Science
140	Terschüren et al. (2009): “Health status of ‘Ruhr-City’ in 2025--predicted disease burden for the metropolitan Ruhr area in North Rhine-Westphalia”	Germany	Various	Mixed data sources	Trend extrapolation (Status quo)	2025	10.1093/eurpub/ckp060	European Journal of Public Health
141	Bagust et al. (2002): “The projected health care burden of Type 2 diabetes in the UK from 2000 to 2060”	GB	Diabetes	Literature review	Trend extrapolation (Status quo)	2060	10.1046/j.1464-5491.19.s4.2.x	Diabetic Medicine
142	Bickel (2002): “Dementia in advanced age: estimating incidence and health care costs”	Germany	Dementia/ Alzheimer’s	Literature review	Trend extrapolation (Status quo)	2050	PMID: 11393002	Zeitschrift für Gerontologie und Geriatrie
143	Morrison et al. (1995): “The impending Canadian prostate cancer epidemic”	Canada	Cancer	Registries	Trend extrapolation	2016	PMID: 7497416	Canadian Journal of Public Health
144	Heidenreich et al. (2013): “Forecasting the impact of heart failure in the United States: a policy statement from the American Heart Association”	USA	Heart failure	Survey	Trend extrapolation (Status quo)	2030	0.1161/HHF.0b013e318291329a	Circulation: Heart Failure
145	Manton & Liu (1984): “Projecting chronic disease prevalence”	USA	Various	Registries	Multistate model(Illness-death mode with recovery)	2000	10.1097/00005650-198,406,000-00002	Medical Care
146	Van Meijgaard et al. (2009): “Assessing and forecasting population health: integrating knowledge and beliefs in a comprehensive framework”	USA	CHD	Mixed data sources	Trend extrapolation	2020	10.1177/003335490912400604	Public Health Reports
147	Bray & Piñeros (2015): “Cancer patterns, trends and projections in Latin America and the Caribbean: a global context”	Various	Cancer	Registries	Trend extrapolation (Status quo)	2030	10.21149/spm.v58i2.7779	Salud Pública de México
148	Evans (1990): “Estimated prevalence of Alzheimer’s disease in the United States”	USA	Dementia/ Alzheimer’s	Other epidemiological studies	Trend extrapolation (Status quo)	2050	PMID: 2233632	The Milbank Quarterly
149	Holman et al. (2011): “The Association of Public Health Observatories (APHO) Diabetes Prevalence Model: estimates of total diabetes prevalence for England, 2010–2030”	GB	Diabetes	Survey	Trend extrapolation	2030	10.1111/j.1464-5491.2010.03216.x	Diabetic Medicine
150	Wille et al. (2010): “Modelling the costs of care of hypertension in patients with metabolic syndrome and its consequences, in Germany, Spain and Italy”	Various	Hypertension	Literature review	Trend extrapolation	2020	10.1007/s10198-010-0223-9	The European Journal of Health Economics
151	Hitzl et al. (2019): “Projected numbers of primary total knee replacement in Austria from 2015 to 2075”	Austria	Joint replacement	Registries	Trend extrapolation (Status quo)	2075	10.1007/s00132-018-3605-9	Der Orthopäde
152	Fox et al. (2011): “Estimating the costs of caring for people with Alzheimer disease in California: 2000–2040”	USA	Dementia/ Alzheimer’s	Literature review	Trend extrapolation (Status quo)	2040	PMID: 11382092	Journal of Public Health Policy
153	Zissimopoulos et al. (2018): “The Impact of Changes in Population Health and Mortality on Future Prevalence of Alzheimer’s Disease and Other Dementias in the United States”	USA	Dementia/ Alzheimer’s	Survey	Trendextrapolation	2040	10.3233/JAD-150233	The journals of gerontology. Series B, Psychological sciences and social sciences
154	Wanneveich et al. (2018): “Impact of intervention targeting risk factors on chronic disease burden”	France	Dementia/ Alzheimer’s	Other epidemiological studies	Multistate model (Illness-death model)	2030	10.1177/0962280216631360	Statistical Methods in Medical Research
155	Vickland et al. (2010): “A computer model of dementia prevalence in Australia: foreseeing outcomes of delaying dementia onset, slowing disease progression, and eradicating dementia types”	Australia	Dementia/ Alzheimer’s	Literature review	Trend extrapolation	2040	10.1159/000272436	Dementia and Geriatric Cognitive Disorders
156	Hooper et al. (2014): “Current trends and projections in the utilisation rates of hip and knee replacement in New Zealand from 2001 to 2026”	New Zealand	Joint replacement	Registries	Trend extrapolation	2026	PMID: 25225759	The New Zealand medical journal
157	Amos et al. (1997): “The rising global burden of diabetes and its complications: estimates and projections to the year 2010”	Global	Diabetes	Literature review	Trend extrapolation (Status quo)	2010	PMID: 9450510	Diabetic Medicine
158	Modig et al. (2012): “The aging population in Sweden: can declining incidence rates in MI, stroke and cancer counterbalance the future demographic challenges?”	Sweden	Various	Registries	Trend extrapolation (Status quo)	2050	10.1007/s10654-012-9653-2	European Journal of Epidemiology
159	Tönnies et al. (2019): “Projected number of people with diagnosed Type 2 diabetes in Germany in 2040”	Germany	Diabetes	Mixed data sources	Multistate model (Illness-death model)	2040	10.1111/dme.13902	Diabetic Medicine
160	Alzheimer Europe (2020): “Dementia in Europe Yearbook 2019 - Estimating the prevalence of dementia in Europe”	Global	Dementia/ Alzheimer’s	Literature review	Trend extrapolation (Status quo)	2050	https://www.alzheimer-europe.org/Publications/Dementia-in-Europe-Yearbooks	Alzheimer Europe

## Abbreviations

CA: Pulmonary, bronchial and tracheal cancer
CHD: Coronary heart disease
COPD: Chronic obstructive pulmonary disease
CVD: Cerebrovascular diseases
HF: Heart failure
SQ: Status quo

## Footnotes

1 See for example the Ageing Report published by the European Commission [8].

2 We chose the year 2060 as the end point of the projection as the official population projection of the German Federal Statistical Office also ends in 2060.

3 This mortality difference can be interpreted as the difference between the mortality rates of the diseased persons ${\boldsymbol{mr}}_{\boldsymbol{a},\boldsymbol{g}}^{\boldsymbol{D}}$ and the population ***mr***_***a***,***g***_ or as the (reverse) difference between the corresponding survival rates ***sr***_***a***,***g***_ and ${\boldsymbol{sr}}_{\boldsymbol{a},\boldsymbol{g}}^{\boldsymbol{D}}$.

4 In the respective year under consideration, we still assume the survival rate of the diseased for the recovered persons before they are transferred to the healthy population in the following year.

5 An exception are age cohorts between 95 and 100 years, whose disease rates were determined in groups because of relatively few data points.

6 The disease-specific input data is determined in the pseudonymised database environment of the AOK Baden-Württemberg via SQL scripts, resulting in only anonymised rates being used for the model calculations. Further calculations are executed using Microsoft Excel.

7 In Germany, this methodology is also used for allocating insureds to risk groups as part of the morbidity-based risk-adjustment scheme in the Statutory Health Insurance [23].

8 Insureds with single diagnoses F34.1 or F38.1 (short depressive episodes) or isolated outpatient diagnosis in the previous year are not excluded from incidence calculation in order to identify new cases with a documented beginning depressive episode in the pre-observation year.

**Table 2:** Diseases and selection criteria

Disease	ICD-10	Validation criteria for outpatient diagnosis
**Arthrosis**	M15-M19	M2Q
**Pulmonary, bronchial and tracheal cancer (CA)**	C33, C34	M2Q
**Coronary heart disease (CHD)**	I20-I25	M3Q and prescription of at least 50 DDD of specific medication^a^
**Chronic obstructive pulmonary disease (COPD)**	J44	M2Q
**Cerebrovascular diseases (CVD)**	I60-I69	M2Q
**Dementia**	F00-F03, F05.1, G23.1, G30, G31.0, G31.82	M2Q
**Depression**	Prevalence: F32, F33, F34.1 Incidence: only F32, not F33 (recurrent depressive disorders)	M2Q
**Diabetes**	E10-E14	M2Q or at least two prescriptions of antidiabetics/blood glucose test strips^b^
**Dorsal pain**	M40-M54	M2Q
**Heart failure (HF)**	I50, I11.0, I13.0, I13.2	M3Q and prescription of at least 50 DDD of specific medication^c^

Source: Own depiction using the ICD-10 classification system and according to the criteria published by AOK Research Institute [19–22]

^a^ ATC groups B01AC, C07, C01D, C08, C09A, C09BB, C09C, C09DB-

^b^ ATC groups A10A, A10B, A10X, V04CA03

^c^ ATC groups C09A, C09B, C07, C01A, C03, C08, C09C, C09D

9 However, for dementia we will assume emerging recovery rates in the scenario *Extended Recovery* for reason of comparability to the other diseases.

10 According to other studies, no mortality difference was found for arthrosis and dorsal pain [25, 26].

11 The group of 0–17-year-olds is left out because the considered diseases are very rare in these cohorts.

12 Age ***a*** is limited between 0 and 100 years and with regard to gender, a distinction is made between male and female cohorts.We model our own population projection as Destatis does not publish a scenario without a future shift in migration. For this purpose, we use the data of mortality.org to model the survival rate for persons older than 100 years and calibrate the data on the life tables publishes by Destatis for the *L2* scenario. In a last step we aggregate the numbers for all persons older than 100 years as our disease specific input data has only few data points for cohorts of age 100 and older.

13 In line with the *W2* scenario published by Destatis, we assume an average positive net migration of 220,000 persons and consider their composition of age groups published by Destatis.

14 The calculations of the studies mentioned must be compared with the results of the scenario *Expansion 1* with consideration of migration, because in all studies the disease rates are also transferred to migrants.

15 For example, Kingston et al. (2018) [33] use a dynamic microsimulation model to project not only prevalence but also the number of diseases per patient and predicted an increase in complex multimorbidity with more than four diseases over the next 20 years.
